# Membrane-Inserting α‑Lipid Polymers: Understanding Lipid Membrane Insertion and Effect on Membrane Fluidity

**DOI:** 10.1021/acs.chemmater.5c00658

**Published:** 2025-07-18

**Authors:** Lorenzo Schiazza, Gokhan Yilmaz, Pavel Gershkovich, Vivien Yeh, Boyan Bonev, Charles Laughton, Snow Stolnik, Giuseppe Mantovani

**Affiliations:** † School of Pharmacy, University of Nottingham, Nottingham NG7 2RD, U.K.; ‡ Department of Chemistry, University of Warwick, Coventry CV4 7AL, U.K.; § Biodiscovery Institute and School of Life Sciences, University of Nottingham, Nottingham NG7 2RD, U.K.

## Abstract

Membrane-inserting materials bearing a lipid residue at one end of their macromolecular chain, α-lipid polymers, are increasingly utilized in biological and pharmaceutical fields. Insertion of these materials into lipid membranes underlines several clinically available liposomal formulations and led to the identification of cellular targets in drug discovery. Herein, we approach this concept from the perspective of a lipid membrane to investigate the relationship between the molecular structure of the inserting α-lipid polymer and the effects that the insertion has on the membrane properties. We synthesized libraries of hydrophilic (co)­polymers comprising neutral or acidic monomers, including *N*-hydroxyethyl acrylamide (HEA), acrylic acid (AA), and 3-acrylamide propanoic acid (3-AAPA), and either of two terminal membrane-inserting moieties with different molecular structures, cholesteryl (Chol) or 1,2-dioleyl-*sn*-glycero-3-phosphoethanolamine (DOPE) phospholipid. We investigated the structure–function relationships combining experimental methods (laurdan generalized polarization, flow cytometry, ^13^C and wide-line ^31^P solid-state NMR, and surface plasmon resonance) in conjunction with in silico modeling. Our data indicate that insertion of α-lipid polymers increases the fluidity of a range of artificial lipid membranes as well as cell plasma membranes in Caco-2 cell culture. The extent of α-lipid polymer–membrane association, described by kinetic and thermodynamic parameters *K*_a_ and *K*_d_, and *K*_D_, respectively, depends on both (i) the nature of the membrane-inserting anchor and (ii) the length of the hydrophilic chain. Hexadecahydro-3H-cyclopenta­[*a*]­phenanthrene-structure-based cholesterol anchor shows faster and stronger membrane association than phospholipid (DOPE) ones. In addition, the shorter polymers (targeted DP = 50 as opposed to DP = 100) display a higher level of membrane association which leads to a consequent larger, from 1.3- to 2.2-fold depending on the polymer, increase of bilayer fluidity. In silico molecular modeling of Chol-(HEA)_n_ polymers suggests that an increase in overall membrane fluidity results from a significant disruption of the lipid organization occurring near the point of insertion of the cholesterol anchor into the membrane. This effect decreases rapidly further away from the insertion point. Our work hence shows that the insertion of α-lipid polymers has a significant effect on lipid bilayer membranes, whereby the observed increase in membrane fluidity needs to be considered when, for example, designing drug formulations or modifying biological systems where it may impact on liposomal membrane stability in biological environment or ability to retain therapeutic cargo.

## Graphical abstract

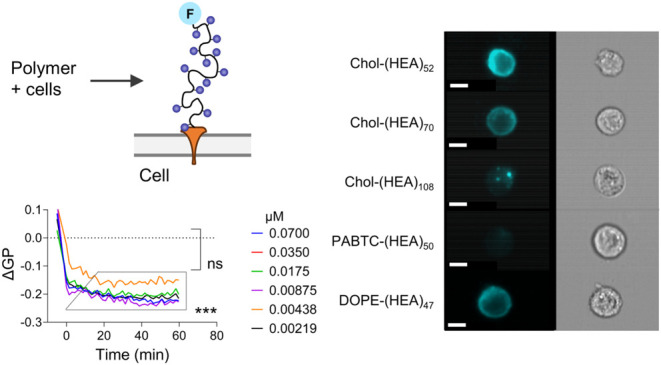

## Introduction

Membrane-inserting synthetic polymers possessing a lipid functionality at one of the polymer chain-ends, named here α-lipid polymers, are increasingly utilized to enhance the efficacy of approved drug formulations,^1,2^ as chemical tools to dissect complex biologic processes^3–5^ and to introduce recognition patterns at the surface of the cell plasma membrane.^6–11^

In the context of pharmaceutical formulations, decoration of liposomal membranes with DSPE-PEG, consisting of mPEG bearing a 1, 2-distearoyl-*sn*-glycero-3-phosphoethanolamine group at one polymer chain-end is utilized, for example, in DOXIL, a clinically used anticancer medicine and Onivyde, a liposomal formulation of irinotecan for the treatment of metastatic pancreatic cancer,^12^ as well as in other PEG surface-modified lipid formulations currently in preclinical nanoparticles. The recently developed COVID-19 mRNA vaccines BNT162b2 and mRNA-1273 lipid nanoparticle formulations include mPEG with lipid chain-ends, namely, 2-mPEG2000-*N*,*N*-ditetradecylacetamide (ALC-0159) and mPEG2000-myristoyl diglyceride (DMG-PEG2000), respectively.^13,14^

Polymers functionalized with lipid end-groups are often utilized to graft specific ligands to nanovesicle formulations.^15^ For example, we recently developed cholesterol-terminated mannosylated glycopolymers and showed that they can enable CD206-mediated uptake of gentamicin-loaded liposomes in IL 4-stimulated RAW 264.7 macrophages and improve eradication of the intracellular pathogen .^16^

Owing to their ability to insert into cell plasma membrane, α-lipid synthetic polymers have been increasingly used as molecular probes to introduce specific functionalities on the surface of target cells or to interrogate complex biological systems. For example, by decorating the surface of human tumor MCF7 and T47D cells with 1,2-dipalmitoyl-rac-glycer-3-phosphoethanolamide-terminated GalNAc glycopolymers, Waver, Bertozzi, and co-workers showed that a bulky glycocalyx promotes a tumor phenotype by increasing integrin adhesion and signaling.^17^ Using α-cholesterylamine glycopolymers as synthetic glycoprotein mimics, the same groups then unveiled the role of integrin mechanosignaling in promoting mesenchymal-like glioblastoma, and induce cancer cell growth, survival, invasion, and treatment resistance.^3^ Subsequent work also uncovered the role of bulky cell glycocalyx in contributing to the formation of metastases by promoting G1 cell cycle progression.^5^

In a landmark study, Bertozzi and co-workers utilized a library of glycopolymers α-functionalized with a 1,2-dipalmitoyl-*sn*-glycero-3-phosphoethanolamine (DPPE) phospholipid to show that artificially sialylated cancer cells are protected from natural killer (NK) cell killing through the engagement of Siglec-7, providing important insight on the link between cancer hypersialylation, clinically observed in many tumors, and immunoevasion.^4^ This uncovered a previously unknown role of Siglec receptors as immune checkpoints^18^ and identified sialome editing as a new strategy for anticancer immunotherapy.^19^ Finally, in separate studies, α-lipid glycopeptides allowed to identify the potential of Siglec-9 agonists to suppress immune cell reactivity^7^ and demonstrated this concept in the context of severe COVID-19, by blocking neutrophil activation and production of neutrophil extracellular traps.^20^

With α-lipid polymer application spanning from the stabilization of clinically used medicines to their use as probes to understand biological pathways and disease processes, here we investigate the effect of the insertion of α-lipid polymers on the physicochemical properties of artificial and cell membranes. For liposomal formulations, membrane fluidity affects stability, interactions with cells,^21,22^ bacteria,^23^ and biological barriers,^24^ as well as their permeability (to their cargo)^25^ and pharmacokinetics.^26,27^ In cells, the fluidity of cellular membranes regulates lateral diffusion of lipids and proteins^28,29^ and hence affects a range of cellular functions, including cell adhesion and migration.^30,31^

Accordingly, we designed three families of α-lipid polymers with two different membrane-inserting α-lipid groups (cholesterol *vs* 1,2-dioleyl-*sn*-glycero-3-phosphoethanolamine (DOPE) phospholipid), hydrophilic repeating units, and macromolecular size. Using both artificial membranes and cells, we show that insertion of α-lipid polymers affects their lipid membrane fluidity, assess the differences in kinetic and thermodynamic binding parameters, and provide a structure–function relationship for the α-lipid polymers investigated. Additionally, molecular modeling provides an *ab initio* understanding of the observed effects of α-lipid polymer insertion on the fluidity of lipid bilayers.

## Materials and Methods

Detailed information, synthesis of MalPyr-fluorescent dye, fluorescent tagging of Chol­(HEA)_n_, DOPE-(HEA)_n_, and PABTC-(HEA)_n_, polymer characterization and cytotoxicity, and instrumentation, is available in the Supporting Information.

### RAFT Polymerizations

In a typical polymerization reaction, the selected RAFT agent was solubilized in DMSO in a Schlenk tube, and then the chosen monomer(s) was (were) solubilized in deionized water (2 M monomer concentration) and added to the DMSO solution (DMSO:water 4:1 vol:vol). ^1^H NMR analysis of an aliquot withdrawn from the reaction mixture before starting the polymerization was carried out to confirm the exact initial RAFT agent:monomer(s) ratio. An appropriate volume of a 2.0 mg mL^–1^ aqueous stock solution of VA-044 radical initiator ([RAFT agent]:[VA-044] = 1:0.025 mol:mol) was added. The tube was then sealed with a rubber septum, cooled to 0 °C, and deoxygenated by N_2_ bubbling for 15 min. The Schlenk tube was then heated to 70 °C, under vigorous stirring. After 2 h, the reaction was stopped by rapid cooling (ice bath) and exposure to air. Finally, the reaction solution was analyzed by ^1^H NMR to determine the final monomer conversion. If the target monomer conversion (>90%) was not reached, another aliquot of VA-044 was added, the reaction mixture was cooled to 0 °C, deoxygenated by N_2_ bubbling for 15 min, and finally heated at 70 °C under stirring for further 2 h.

To purify the polymers from unreacted monomers and reagents, the viscous reaction mixtures were diluted with CH_2_Cl_2_:MeOH 6:5 vol:vol and precipitated (in acetone for Chol- and PABTC-terminated polymers, or *tert*-butyl methyl ether for DOPE- terminated polymers) in a Falcon tube. The suspension was centrifuged (4500 rpm, 10 min), and the supernatant was removed. The solid residue was dried under reduced pressure, then suspended in water, and purified by dialysis against 4 L of deionized water for 2–3 days, changing the solvent every 12–24 h. The dialysis was carried out using an appropriately sized MWCO membrane (1, 2, 5, or 7 kDa) and finally the polymer solutions were freeze-dried.

The degree of polymerization (DP) of the isolated polymers was estimated by ^1^H NMR, by comparing the integrals of specific polymer α-groups and those of the CH_2_ and CH residues of the polymer backbone (1.6 and 2 ppm, respectively). To estimate DP of polymers synthesized with DOPE-RAFT agent **(2)**, the signals for the DOPE CH_3_ groups at 0.78 ppm were used as internal reference. For polymers synthesized with cholesterol RAFT agent **(1)**, the signal used as reference was a cholesterol CH_3_ triplet at 0.64 ppm. For the PABTC RAFT agent **(3)**, the signal used as a reference was that of CH_3_ at 0.93 ppm. Characterization data for all synthesized polymers are shown in Table S1. SEC traces are shown in Figures S2–S5.

### Laurdan Assay

Caco-2 cells were seeded at a concentration of 1 × 10^4^ per well in 96-well plates (black) and cultured for 24 h. The medium was removed from the cells and these were incubated with a 2 μM solution of laurdan dye in HBSS for 30 min.^32^ Following exposure, the dye was washed off twice with PBS, and then cholesterol-, DOPE-, and non-membrane-inserting PABTC-terminated control polymers at the chosen concentration(s) in HBSS (150 μL) were added to the wells. After 30 min of incubation, fluorescence was measured at 360/460 nm (λ_ex_/λ_em_) and 360/490 nm (λ_ex_/λ_em_), at 37 °C.

Generalized polarization (GP) of Laurdan was calculated by subtracting readings for the blank samples (Caco-2 cells + medium, to account for cell autofluorescence) to the values measured for each well containing cells and Laurdan, using the following formula (eq S1):

$$\mathrm{GP}=\frac{\left(I_{460}-I_{490}\right)}{\left(I_{460}+I_{490}\right)}$$

where I_460_ and I_500_ represent fluorescence emission intensities at λ_em_ = 460 and λ_em_ = 490 nm, respectively, following excitation at λ_ex_ = 360 nm.

The 96-well plates were analyzed with a Spark 10 M Plate Reader and data were recorded using the SparkControl 2.1 software. Data were processed with Prism 9.4.1 (GraphPad).

### Flow Cytometry of Caco-2 Cells Treated with MalPyr Blue-Tagged (HEA)_n_ Polymers

Caco-2 cells were seeded at a concentration of 1 × 10^4^/mL in 12-well plates in phenol red-free EMEM. After 24 h incubation, the medium was removed from the cells, and these were treated with an appropriate volume (1 mL) of the fluorescently tagged polymer solutions (0.1 μM in HBSS) for a 20 min incubation. The fluorescent polymer solutions were removed, and cells were washed twice with PBS to remove the polymer solutions. The cells were exposed to an appropriate quantity (0.3 mL per well) of a prewarmed (37 °C) trypsin-EDTA solution. Cells were incubated for 5 min in the standard cell culture conditions. Then, an equal amount (0.3 mL) of prewarmed phenol red-free medium was added to the detached cells, to inhibit further effects of trypsin and to recover as many cells as possible. The cell suspensions were transferred into 1.5 mL Eppendorf vials for centrifugation (250 g for 4 min), to obtain a pellet of cells. The supernatant was aspirated, and the cell pellet was suspended in 40 μL of room temperature PBS. Cells were stored on ice and covered from light for 2 h, and then were analyzed on an Amnis ImagestreamX MKII Imaging Flow Cytometer (Luminex). The images and data were elaborated using the IDEAS 6.0 software (LuminexCorp). The results are expressed as adjusted median fluorescence intensity (aMFI), calculated as aMFI = (median fluorescence intensity (sample))/(median fluorescence intensity (untreated cells) × tagging efficiency). Flow cytometry images are shown in Figures S11–S15.

### Surface Plasmon Resonance (SPR) Analysis

The interaction between cholesterol-, phospholipid DOPE, and non-membrane-inserting PABTC-terminated polymers and immobilized lipid bilayers was performed on a BIAcore T200 system (GE Healthcare). The SPR measurements were carried out after depositing an immobilized lipid bilayer by passing liposomes on a BiaCore L1 chip (Cytiva Life Sciences, Sheffield, UK).

Solutions of selected polymers were prepared at different concentrations (24–1.5 nM) in HBSS buffer solution. Sensorgrams for each polymer concentration were recorded with a 240 s injection of polymer solution (ON period) followed by 120 s of buffer alone (OFF period). Kinetic data was processed using a single set of sites (1:1 Langmuir binding) model in the BIAevalulation 3.1 software.

#### Liposome Preparation

Liposomes were prepared following a gentle hydration method,^33,34^ followed by membrane extrusion.^35^ Briefly, phospholipid stock solutions (2.5 mg/mL in CH_2_Cl_2_) were used to solubilize solid cholesterol in a round-bottomed flask, to achieve a DOPC:DSPC:cholesterol 2:1:1 mol:mol:mol ratio. The resulting organic solution was diluted with a CH_2_Cl_2_:MeOH 2:1 solution to reach a final 10 mg mL^–1^ lipid concentration. The organic solution was dried under reduced pressure using a rotary evaporator. The dried lipid film was heated to 55 °C and preheated (55 °C) HBSS buffer was added dropwise while stirring, to achieve a 0.02 mg mL^–1^ lipid concentration. The mixture was stirred gently (100 rpm) until it became clear and then was subjected to 21 successive extrusions through a polycarbonate membrane (0.2 μm pore size). The resulting liposome suspension was analyzed by DLS for particle size analysis (Figure S17).

The loading process was conducted in three successive steps:

Surface wash: The L1 chip surface was cleaned with repeated (at least 2) injections of 10 μL of 20 mM CHAPS detergent solution.

Liposome loading: The liposome suspension (prepared as described above) was injected at a low flow rate (5 μL min^–1^) at a 0.5 mM lipid concentration. Adsorption was monitored by following an increase in Response index Units (RiU), which plateaued as the surface coverage approached completion. Previous studies by Karlsson^36^ and Kamimori^37^ suggest that values around 5000 RiU are indicative of the formation of a supported bilayer on the sensor surface. Accordingly, the liposome suspension (0.02 mg/mL in 10 mM HBSS) was then injected on the L1 chip at 5 μL min^–1^, until a stable baseline at 5600 RiU was reached. Bilayer immobilization was found to be complete within a few minutes.

Liposomal surface cleaning: Regeneration of the sensor chip surface was performed using a long pulse of the buffer solution (10 mM HBSS) at high flow rate (50 μL min^–1^, 420 s).

### Solid-State NMR Analysis of Multilamellar Lipid Vesicles

The selected polymers were solubilized or suspended in the minimum amount of deionized water and added to solid dimyristoylphosphatidylcholine (DMPC:polymer 10:1 molar ratio); the mixture was stirred until an almost clear suspension was obtained. The mixture was subjected to 8 freeze–thaw cycles to produce multilamellar vesicles and then centrifuged for 20 min at 1500 rpm. The supernatant was removed, and the pellet was collected and transferred to the solid-state NMR probe. All solid-state NMR experiments were performed with a Varian 400 MHz VNMRS spectrometer equipped with a 4 mm magic angle spinning (MAS) NMR probe. ^31^P and ^13^C NMR spectra were referenced externally with 10% H_3_PO_4_ and adamantane, respectively.

^31^P wide-line NMR was performed at 28 °C using a Hahn echo sequence with a 100 kHz π/2 pulse and 12 μs delays. Spectra were acquired with 20 ms acquisition time with a recycle delay of 5 s and 2048 transients averaged. ^13^C magic angle spinning (MAS) NMR was performed at an MAS frequency of 5 kHz and at 32 °C, for both cross-polarization and direct excitation experiments. Direct excitation experiments were done with a single 100 kHz π/2 before acquisition, followed by 125 ms acquisition under 60 kHz SPINAL-64 to remove heteronuclear dipolar couplings. The recycle delay was 5 s, and spectra were obtained after an average of 2048 transients.

Cross-polarization was performed with an initial 120 kHz π/2 1H excitation pulse, followed by 3.5 ms of 45 kHz Hartmann–Hahn contact time for magnetization transfer to 13C. Spectra were recorded under a 60 kHz SPINAL-64 scheme over 125 ms of acquisition time. The recycle delay was 3.5 s, and spectra were obtained by averaging 2048 transients.

### Molecular Modeling of the Interactions of Chol-HEA_n_ Homopolymers with a DPPC Bilayer Membrane

All simulations were performed using an AMBER 2028–31. For atomistic modeling purposes, Chol-(HEA)_n_ polymers were divided into cholesterol, linker, HEA repeating units (both enantiomers), and terminal units, with appropriate capping groups. Models for each were built using Chimera,^38^ and then unit parameter files generated using AMBER’s Antechamber module.^39^ Calculated mean membrane thickness (minimum distance between phosphorus atoms in the two leaflets) as a function of radial (x,y) distance from the inserted cholesterol for the insertion is shown in Figure S22.

## Results and Discussion

### Synthesis of α-Lipid Polymers

First, a library of polymers was produced, where (i) the nature of the membrane-inserting α-group, (ii) the nature of hydrophilic repeating units, and (iii) the average chain length were systematically varied. These materials were synthesized by reversible addition–fragmentation chain-transfer polymerization (RAFT),^16,18^ which allows for specific functional groups to be placed at the α end of polymer chains, while leaving a thiocarbonylthio group at the other (omega) chain-end available for further chemical modification, if required. The latter feature was exploited in this work to fluorescently tag a subfamily of membrane-inserting polymers to enable tracking in cell studies (*vide infra*). This study focuses on materials with cholesterol as the membrane-inserting α-groups, with a smaller library of polymers with 1,2-dioleyl-*sn*-glycero-3-phosphoethanolamine (DOPE) phospholipid, possessing two monounsaturated oleoyl ester groups, that were synthesized and used for comparative purposes. The latter materials were synthesized from a DOPE-based RAFT agent containing a butyl-trithiocarbonate moiety in its Z group. Control polymers were prepared with a non-membrane-inserting 2-propanoic acid as a hydrophilic chain-end, using propanoic acid-2-[[(butylthio)­thioxomethyl]­thio]-carbonate (PABTC) as the RAFT agent. Owing to the high hydrophobic character of cholesterol, a RAFT agent containing a hydrophilic propanesulfonate in its Z group^16^ was chosen to synthesize our cholesterol (Chol)-terminated hydrophilic polymers under polar reaction conditions. The structures of all RAFT agents are shown in Chart S1.

The hydrophilic monomers chosen for this study are *N*-(2-hydroxyethyl)­acrylamide (HEA), acrylic acid (AA), and 3-acrylamide propanoic acid (3-AAPA) (Figure 1b). Neutral hydroxyethyl acrylamide monomer was selected because of the reported biocompatibility of the resulting polymers,^40,41^ while acrylic acid and 3-acrylamide propanoic acid were chosen to assess the effect of polymer polar acid functionalities on the physicochemical properties of lipid membranes following polymer insertion. All homo- and copolymers were prepared using a modified version of Perrier’s fast RAFT polymerization method.^42^ In this initial library of polymers, degree of polymerization (DP) of approximately 50 and 100 were targeted. Polymer characterization, size exclusion chromatography (SEC) data (weight and number-average molar masses, as well as dispersity values), for the synthesized polymers is shown in Table S1 and Figures S1–S4.

**Figure 1:**
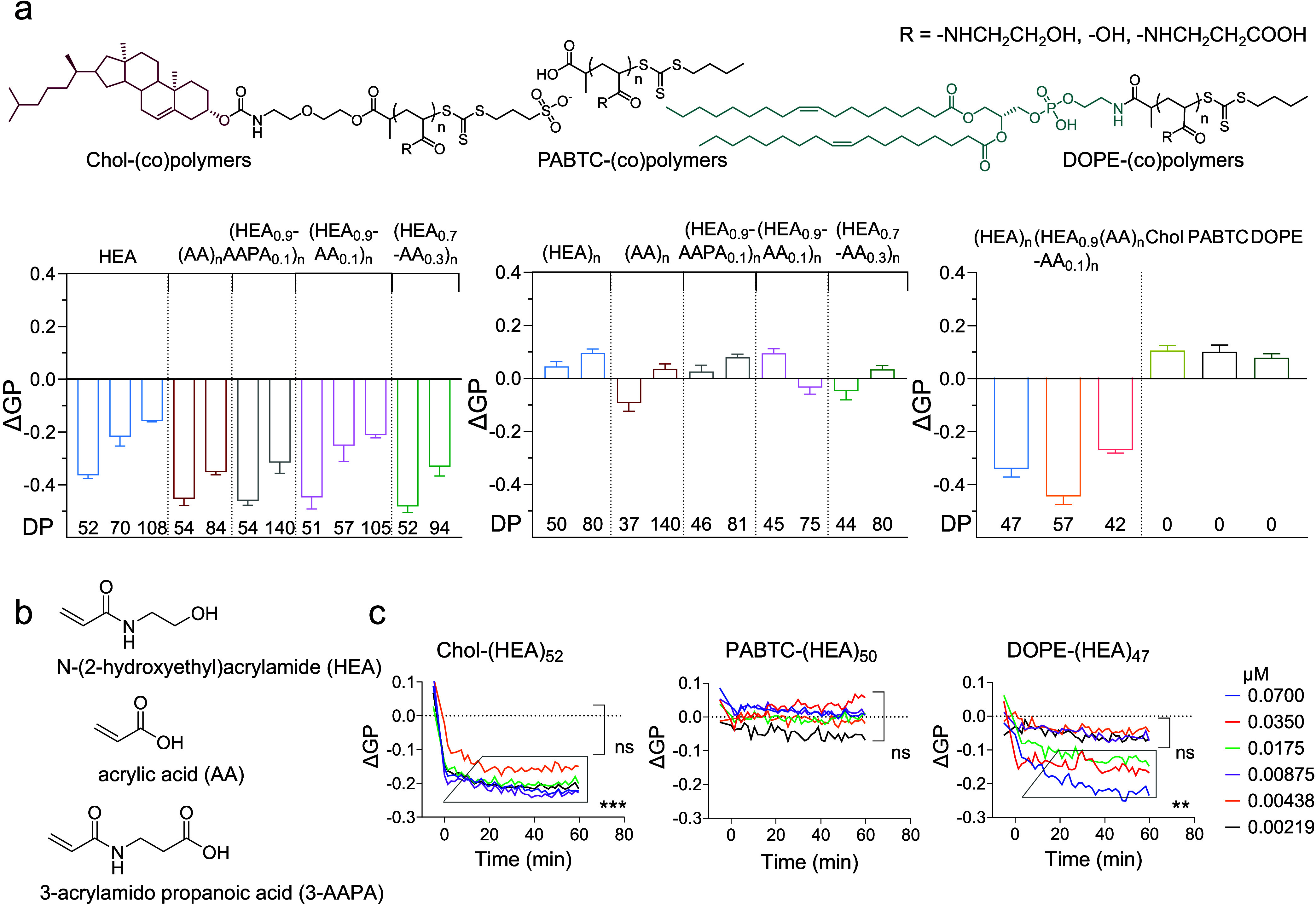
Effect of membrane-inserting polymers on fluidity of cell plasma membrane, as assessed by laurdan assay. (a) *Top*: chemical structure of cholesterol (Chol)-, propanoic acid-2-[[(butylthio)­thioxomethyl]­thio]-carbonate (PABTC)- and 1,2-dioleyl-*sn*-glycero-3-phosphoethanolamine (DOPE) phospholipid-containing homo- and random copolymers utilized in this work. PABTC-containing polymers were used here as non-membrane-inserting control materials. *Bottom*: Laurdan membrane fluidity assay: Caco-2 cells were incubated for 30 min with 0.1 μM Chol-, DOPE-, and non-membrane-inserting PABTC-terminated control polymers. Low molecular weight RAFT agents (Chol, PABTC, and DOPE, structures in Chart S1), were also tested (right panel). Polymer name codes indicate both the relative proportion of the different repeating units and the total number of repeating units (*n*, also indicated as DP, that is, the degree of polymerization of the tested polymers). For example, (HEA_0.9_-AA_0.1_)_n_ indicates a polymer that has a total number of repeating units of “*n*”, 90% of which come from *N*-(2-hydroxyethyl)­acrylamide (HEA) and 10% from acrylic acid (AA). DP (*n*) of each polymer is reported under each bar column. Results are expressed as delta generalized polarization (ΔGP), which is here the difference between the cells incubated with polymers and that of untreated cells. A positive value represents an increase in membrane rigidity, while a negative value represents an increase in membrane fluidity, compared with untreated cells. (b) Chemical structure and abbreviations for monomers utilized to synthesize the materials used in this study. (c) Membrane fluidity kinetic assay (assessed by laurdan assay on Caco-2 cells, at variable polymer concentration, incubation time: 60 min). Statistical significance of the results was assessed through a one-way ANOVA test, comparing the fluidity values obtained with the values measured for untreated cells (** *P* < 0.01, *** *P* < 0.001, *n* = 2).

The hydrophilic monomers chosen for this study are *N*-(2-hydroxyethyl)­acrylamide (HEA), acrylic acid (AA), and 3-acrylamide propanoic acid (3-AAPA) (Figure 1b).

Neutral hydroxyethyl acrylamide monomer was selected because of the reported biocompatibility of the resulting polymers,^40,41^ while acrylic acid and 3-acrylamide propanoic acid were chosen to assess the effect of polymer polar acid functionalities on the physicochemical properties of lipid membranes following polymer insertion. All homo- and copolymers were prepared using a modified version of Perrier’s fast RAFT polymerization method.^42^ In this initial library of polymers, degree of polymerization (DP) of approximately 50 and 100 were targeted. Polymer characterization, size exclusion chromatography (SEC) data (weight and number-average molar masses, as well as dispersity values), for the synthesized polymers is shown in Table S1 and Figures S1–S4.

### Chol- and DOPE-Terminated Polymers Increase the Fluidity of Caco-2 Cells Plasma Membrane

In this study, we first assessed the effect of all synthesized Chol-, DOPE-, and PABTC-terminated polymers on the membrane fluidity of Caco-2 cells, a cell line routinely used as a model for intestinal epithelium,^43^ and then we investigated the modality of polymer–membrane interactions at a molecular level using a selected subgroup of these α-lipid polymers. Accordingly, the effect of these polymers on lipid bilayer membrane fluidity was first assessed on Caco-2 cells using a laurdan assay,^44^ a membrane fluorescent dye sensitive to local membrane packing. Results are expressed as the difference between the generalized polarization (GP, eq S1) of polymer-treated vs. untreated cells, where positive values are indicative of rigid lipid bilayers, while negative ones are suggestive of more fluid lipid membranes. Results in Figure 1a,c summarize data on exposure of Caco-2 cells, pretreated with laurdan, to α-lipid polymers at a concentration of 0.1 μM (0.5–1.5 mg mL^–1^). Data are reported as ΔGP, which is the generalized polarization relative to the values for untreated cells.

Cholesterol-containing α-lipid polymers resulted in a negative ΔGP (spanning from −0.16 to −0.46), indicating an increase in cell membrane fluidity (Figure 1a). Interestingly, shorter α-lipid polymers increase membrane fluidity more than their corresponding analogues with longer hydrophilic chains, i.e., ΔGP trend follows: Chol-(HEA)_52_ < Chol-(HEA)_70_ < Chol-(HEA)_108_. DOPE-based α-lipid polymers show a similar trend in increasing the fluidity of Caco-2 cell membrane (spanning from −0.27 to −0.44). These results are in agreement with previous studies conducted using poloxamers amphiphilic block copolymers, showing that polymers with sufficiently large hydrophobic blocks interact with cell plasma membranes, increasing their fluidity.^45,46^ More in general, through the formation of lipid-polymer composite materials, amphiphilic block copolymers have been used to modify a range of physicochemical properties of lipid membranes,^47–58^ including their fluidity. Interestingly, our work shows that for α-lipid polymers, a small terminal hydrophobic residue, i.e., Chol– or DOPE–, is sufficient to induce lipid bilayer fluidization.

Conversely, control polymers lacking α-lipid end-groups (PABTC based) had minimal effect on cell membrane fluidity, suggesting that the [[(butylthio)­thiomethyl]­thio]-carbonate at their ω chain-end was either not able to insert into the membrane or that such insertion, if it occurred, had very minimal effect on cell membrane fluidity. In a similar manner, the effect of Chol-, PABTC-, and DOPE-RAFT agents, structurally analogous to the polymer chain-ends but lacking the hydrophilic polymer chain, also had a minimal effect on membrane fluidity. Finally, the presence of acidic functionalities (acrylic acid (AA) and 3-acrylamide propanoic acid (3-AAPA)) within the polymer chain did not significantly affect the observed ΔGP, showing that, for the materials investigated, the nature of the hydrophilic repeating units had no significant effect on the ability of these α-lipid (co)­polymers to induce membrane fluidization.

In terms of kinetics of membrane insertion for α-lipid polymers with Chol and DOPE-terminal groups, data showed that for Chol-(HEA)_53_, the decrease of Caco-2 plasma membrane fluidity (more negative ΔGP) occurs rapidly, reaching near-maximal effect within a few seconds, while for DOPE-(HEA)_47_, near-maximal effect on ΔGP was only reached after approximately 30 min (Figure 1c). As expected, for both materials, the effect on membrane fluidity was found to be concentration-dependent. In agreement with the initial laurdan assay experiments (Figure 1a), no significant effect on membrane fluidity was observed for control polymer, PABTC-(HEA)_50_, which lacks α-lipid anchors able to insert into lipid bilayers (Figure 1c).

To assess whether the laurdan assay results could be ascribed, at least in part, to polymer cytotoxicity or polymer-induced plasma membrane damage, the effect of these materials on cell metabolic activity and membrane integrity was tested by PrestoBlue and LDH assays, respectively. In initial experiments, Caco-2 cells were incubated with Chol-, DOPE-, and PABTC-polymers in the 0.02–0.1 μM range of concentrations for 24 h. Interestingly, most cholopolymers induced an increase in cell metabolic activity, irrespective of their monomer composition (Figure S17). This effect could be the result of a stress-induced survival response, as we previously described for Caco-2 cells treated with nonionic surfactant Kolliphor HS15.^32^ Conversely, with the exception of phospholipid DOPE-terminated polymers (Figure S19), concentration-dependent LDH release was observed for most polymers due to membrane instability induced by these materials, while cells were still metabolically viable (Figures S17 and S18). However, when incubated for 30 min, the duration of the cell membrane fluidity laurdan assay, the polymers that showed the highest increase in cell metabolism and LDH levels in our initial screening, Chol-[(HEA)_0.9_-*r*-(AA)_0.1_]_57_ and Chol-[(HEA)_0.7_-*r*-(AA)_0.3_]_54_, had no effect on cell metabolism (PrestoBlue) and membrane integrity (LDH) (Figure S20). Finally, the LDH essay was then repeated with Chol- and PABTC-polymers at the highest tested concentration (0.10 μM) and 30 min incubation time for a wider range of Chol- and control PABTC-polymers. Results showed that under these conditions, these materials did not induce any LDH release (Figure S21), and thus did not negatively affect cell membrane permeability. Together, these results indicate that, for the cell-polymer exposure time utilized in these experiments, the effect of polymers on the membrane fluidity of Caco-2 cells was not caused by cytotoxicity or polymer-induced plasma membrane damage.

Taken together, results from this part of the study indicate that (i) Chol- and phospholipid DOPE-terminated polymers increase the fluidity of Caco-2 cells plasma membrane and that this effect is more prominent for polymers with shorter hydrophilic macromolecular chain (DP ∼ 50) than longer ones (DP ∼ 100), as shown by general polarization (GP) data obtained by laurdan assay; (ii) the observed membrane fluidization effect depends on polymer concentration and appears to be faster for polymers with a cholesterol (Chol-) chain-end than for those with a DOPE phospholipid one; (iii) under the conditions utilized for the laurdan assay (0.10 μM polymer concentration and 30 min incubation time), the membrane fluidization effect does not appear to be a consequence of polymer-mediated changes in cell metabolic activity and membrane integrity.

### Probing Chol- and DOPE-(HEA)_n_ Polymers Association with Artificial and Cell Membranes

Next, the association of polymers with Caco-2 cells was assessed using fluorescently tagged (HEA)_n_ cholesterol homopolymers (Chol-(HEA)_108_, Chol-(HEA)_70_, Chol-(HEA)_53_), DOPE-terminated DOPE-(HEA)_47_, and control polymer PABTC-(HEA)_50_. These fluorescently tagged polymers were generated by first converting the trithiocarbonate moiety at the polymer ω chain-end into a sulfhydryl functionality by aminolysis, which was then reacted with the thiol-reactive fluorescent dye MalPyr (Scheme S3).

Organic fluorophores typically contain hydrophobic conjugated systems; thus, here it was important to utilize one that was sufficiently hydrophilic to prevent potential interactions with the cell membrane lipid bilayer. For example, Parthasarathy and co-workers showed that the chemical structure of some fluorophores can drive the association of fluorescently tagged hydrophilic polymers with lipid bilayers.^8^ To this aim, here maleimide-containing *O*-alkyl pyranine (MalPyr, Figure 2a) was designed and synthesized (Scheme S2). MalPyr contains a pyranine fluorophore with three sulfonic acid salt groups, which is analogous to anionic dyes, such as Lucifer Yellow, which are known for their inability to interact with and diffuse through cell membranes.^59^

**Figure 2:**
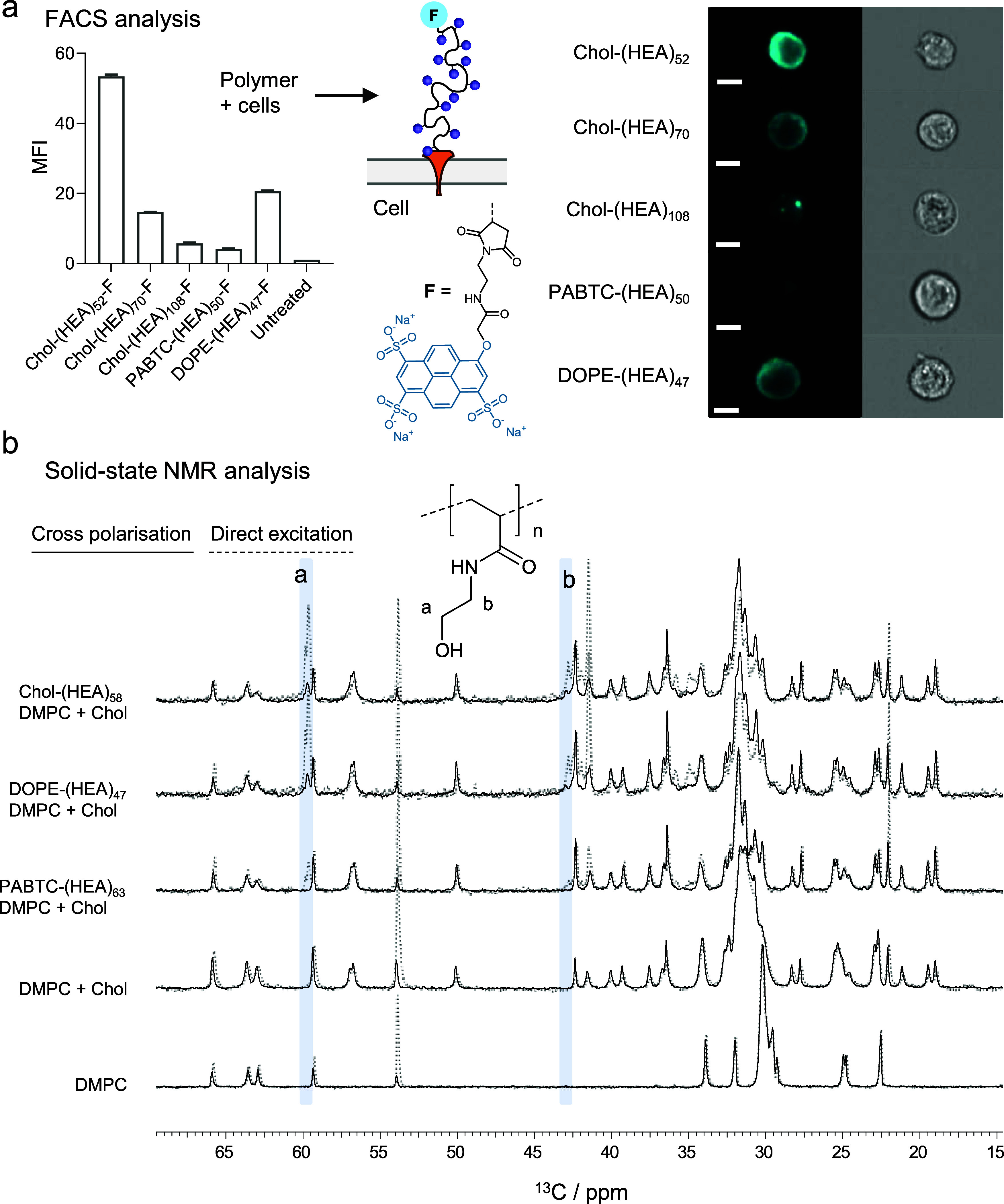
α-Lipid polymers insert into both the cell plasma membrane and the artificial lipid bilayers. (a) Insertion of cholesterol- and DOPE-terminated (HEA)_n_ polymers in the plasma membrane of Caco-2 cells depends on polymer chain length and nature of polymer chain-end. Caco-2 cells were incubated for 20 min with 0.1 μM polymer solutions and analyzed by imaging flow cytometry (FACS). Polymers are fluorescently tagged at the ω-end with *O*-alkyl pyranine *F* = MalPyr; λ_ex_ = 377 nm, λ_em_ = 420 nm (Scheme S3). Scale bars: 10 μm. (b) ^13^C solid-state magic angle spinning (MAS) NMR (ssNMR) confirms binding of cholesterol- or DOPE-terminated (HEA)_n_ homopolymers to immobilized DMPC:cholesterol model lipid bilayer (DMPC:cholesterol 1:1 mol:mol), comparing cross-polarization (CP) and direct excitation. Solid lines are spectra performed with cross-polarization, and dotted lines are spectra performed with direct excitation. Light blue panels highlight characteristic resonances for the HOCH_2_ and CH_2_NH groups of the (HEA)_n_ polymers. The spectra intensities are normalized to the signal at 70.5 ppm, corresponding to the glycerol CH resonance of DMPC.

Accordingly, Caco-2 cells were incubated with MalPyr-tagged polymers (0.1 μM in HBSS) for 30 min, and then the cells were analyzed by imaging flow cytometry (λ_ex_ = 377 nm and λ_em_ = 420 nm). Previous work by the Bertozzi group showed that, over several hours following initial binding to cell membranes, cholesterol- and phospholipid-terminated polymers are slowly but permanently internalized by cells through a membrane lipid recycling mechanism, with the notable exception of lipids such as CholA, which can recycle to the cell membrane.^9^

However, in our work, cells were analyzed immediately after a short incubation (30 min) with MalPyr-fluorescent polymers, before detectable cell internalization could occur. As a result, MalPyr-fluorescent polymers were, therefore, found to be localized at the cell membrane (Figures 2a and S16). For Chol-(HEA)_n_ homopolymers, cell association decreased significantly with the increase in chain length, in the order MalPyr-Chol-(HEA)_52_ > MalPyr-Chol-(HEA)_70_ > MalPyr-Chol-(HEA)_108_ (Figures 2a and S11–S15), indicating the role of hydrophilic chain length in determining the efficiency of membrane insertion. This trend is in line with that observed in the membrane fluidity experiments, where when materials were compared at the same molar concentration of polymer chains, shorter cholesterol-containing polymers induced the largest increase in membrane fluidity (decrease in ΔGP). This suggests that the higher membrane fluidization ability of shorter polymers may be due, at least in part, to a larger number of polymer chains that can insert into cell membranes. In comparison, phospholipid-terminated polymer MalPyr-DOPE-(HEA)_47_ showed significantly lower membrane association compared with its cholesterol-terminated analogue MalPyr-Chol-(HEA)_52_. The MalPyr-PABTC-(HEA)_50_ control polymer showed minimal interaction with Caco-2 cells, in line with the results obtained in the laurdan fluidity assay (Figure 1a).

To investigate the association of these materials with artificial lipid bilayers, we used high-resolution ^13^C solid-state magic angle spinning (MAS) NMR (ssNMR). To facilitate interpretation of spectra here and in subsequent solid-state wide-line ^31^P NMR analysis (*vide infra*), the membrane lipid composition was kept simple, comprising phospholipid (1,2-dimyristoyl-*sn*-glycero-3-phosphocholine, DMPC) and cholesterol which ensured lamellar phase, i.e., lipid bilayer formation at DMPC:cholesterol 1:1 molar ratio.

Accordingly, we compared spectra obtained by direct excitation and cross-polarization (CP), the two different methods for creating observable magnetization in high-resolution ^13^C MAS ssNMR. In cross-polarization, initial proton excitation is transferred to bonded ^13^C nuclei through heteronuclear dipolar coupling, resulting in an enhancement of ^13^C signal due to the higher polarization of ^1^H compared with ^13^C and faster proton longitudinal relaxation.^60^ Cross-polarization ^13^C MAS NMR can be used to study molecular interactions with membranes, due to the sensitivity of this approach to changes in molecular mobility on association with lipid membranes.^61,62^ During CP, the NMR signal is generated through ^13^C magnetization transfer from ^1^H to ^13^C that relies on nuclear dipolar couplings, only present in motionally restricted molecular environments. As a result, the CP MAS NMR signal arises solely from membrane-associated molecules, while macromolecules in solution undergo rapid dynamics and can only contribute to direct excitation measurements.

In our study, polymers associated with the anisotropic membrane environment are observed in CP MAS spectra, while polymers in solution remain undetected. Direct excitation spectra, in contrast, have contributions from both polymer chains in solution and membrane-associated polymers. Figure 2b shows ^13^C CP MAS NMR spectra from DMPC:cholesterol membranes alone and following incubation with HEA_n_ polymers with comparable chain length, Chol-(HEA)_58_, DOPE-(HEA)_47_, and control polymer PABTC-(HEA)_63_. As expected, little difference was observed between direct excitation and CP spectra from DMPC:cholesterol and DMPC-only membranes, as all lipids are organized in the bilayer phase, where cooperative anisotropic molecular dynamics contributes to spectral intensity in both experiments. By contrast, spectra from polymer-containing systems highlight differences between polymers that partition into membranes and polymers that are unable to do so and remain in solution.

Direct excitation from membranes containing PABTC-(HEA)_63_, DOPE-(HEA)_47_, or Chol-(HEA)_58_ showed strong polymer signals, absent from DMPC:cholesterol membranes alone, in particular, the signals at 43 and 60 ppm, arising from the C*H*_2_N and OC*H*_2_ units in the polymer pendant groups, respectively (Figure 2b). The presence of polymer signals in the direct excitation ^13^C MAS NMR spectra simply confirms the presence of polymer and lipid in the sample, irrespective of their putative interactions.

Conversely, these signals are absent in the ^13^C CP MAS spectrum of the control PABTC-(HEA)_63_ polymer incubated with lipid membranes, indicating that PABTC-(HEA)_63_ remains soluble and does not insert within the DMPC:cholesterol membrane. By contrast, the ^13^C CP spectra of DOPE-(HEA)_47_ and Chol-(HEA)_58_ incubated with lipid membranes show signals at 43 and 60 ppm, which arise specifically from the membrane-embedded polymer. The ^13^C CP signal intensity at these chemical shifts is lower than from direct excitation, potentially indicating that a proportion of the polymer remains in solution or that membrane-associated polymer retains relatively high mobility. The results suggest that both DOPE-(HEA)_47_ and Chol-(HEA)_58_ polymers, containing phospholipid DOPE and cholesterol chain-ends, respectively, can intercalate into the model DMPC:cholesterol membrane. Again, control polymer PABTC-(HEA)_63_ did not show membrane interaction as no immobilized polymer resonances were observed in the cross-polarization spectrum, in line with what was observed in both the laurdan assay (Figure 1a) and flow cytometry (Figure 2a), using Caco-2 cells. In future work, if lateral partitioning of sterols were to occur in the membrane, it would be further possible to assess preference of anchored polymers for ordered or disordered subphases by following the segmental orientational order parameter S_CH_ through a combination of direct excitation, INEPT transfer, and CP^63^ or using R18_1_^7^ symmetry-based sequences.^64^

Taken together, data from the second part of our study show that (i) Chol-(HEA)_n_ polymers insert into Caco-2 cells plasma membrane, and cell binding is inversely associated with polymer chain length, i.e., Chol-(HEA)_52_ > MalPyr-Chol-(HEA)_70_ > MalPyr-Chol-(HEA)_108_, as shown by flow cytometry; (ii) extent of cell membrane binding appears to be dependent on the nature of the lipid anchor. For polymers with comparable hydrophilic chain length, Chol-(HEA)_52_ and DOPE-(HEA)_47_, the former gave ∼2.5 times higher cell association than the latter; (iii) through comparison of direct excitation and cross-polarization spectra, ^13^C solid-state NMR enables direct characterization of binding of Chol- and DOPE-(HEA)_n_ polymers to artificial lipid bilayer membranes.

### Effect of Polymer Structure on Lipid Bilayers Association and Fluidity: Kinetic and Thermodynamic Parameters of Binding and Effect on Bilayer Lipid Dynamics

The extent by which molecules bind to lipid membranes has been investigated using either partition coefficients or binding constants as the descriptors of solute/nonpolar medium interactions, and the advantages and limitations of both approaches have been discussed.^65^ Surface plasmon resonance (SPR) has been employed to estimate dissociation constants (*K*_D_) and quantify kinetics of binding of low molecular weight molecules^66,67^ and proteins^68^ to immobilized lipid bilayer membranes.

Several of such studies utilized carboxymethylated dextran coated with hydrophobic alkyl groups (L1 sensor chip)^55–57^ onto which lipids are captured in the form of liposomes, which then undergo structural rearrangement at the surface of the chip to form an immobilized phospholipid bilayer.

Castanho and co-workers confirmed that liposomes trapped on L1 chips can form an immobilized membrane over a wide range of lipid compositions through a combination of confocal fluorescence microscopy imaging and FRAP experiments.^69^ To the best of our knowledge, there are no previous examples of studies where SPR is used to assess the binding of hydrophilic polymers possessing a hydrophobic chain-end to lipid bilayers.

Here, the L1 SPR chip was coated with a phospholipid bilayer formed by 2:1:1 = [DOPC]:[DSPC]:[cholesterol], a lipid system that has been thoroughly characterized in terms of its physicochemical properties.^70^ For these experiments, 2-hydroxyethyl acrylamide homopolymers, Chol-(HEA)_58_, Chol-(HEA)_70_, Chol-(HEA)_108_, DOPE-(HEA)_80_, and PABTC-(HEA)_63_, in the 1.5–24 nM range of concentrations were utilized. The dissociation constant, *K*_D_ = *K*_d_/*K*_a_, was estimated from SPR measurements to compare their relative ability to insert into and retain with immobilized lipid bilayers. All polymers were tested at concentrations at least 1 order of magnitude below their critical aggregation concentration (CAC) (Figure S8) to prevent the formation of supramolecular aggregates in equilibrium with individual polymer chains, which could potentially affect the assessment of kinetic and thermodynamic binding parameters.

In these SPR experiments, the tested cholesterol-terminated polymers Chol-(HEA)_58_, Chol-(HEA)_70_, Chol-(HEA)_108_ gave very similar dissociation constants *K*_D_, which were all in the subnanomolar range (Figure 3a and Table S3). Conversely, phospholipid-terminated DOPE-(HEA)_80_ had a higher dissociation constant *K*_D_ (1.5 × 10^–8^ M) compared with its cholesterol-terminated polymer counterpart with a similar chain length Chol-(HEA)_70_ (*K*_D_ = 8.9 × 10^–10^ M). *K*_D_ is a thermodynamic dissociation constant; thus, higher values indicate a more favorable dissociation of the polymers from the immobilized lipid bilayer. Interestingly, DOPE-(HEA)_80_ showed significantly different kinetic profiles compared with cholesterol-terminated Chol-(HEA)_70_. Indeed, DOPE-(HEA)_80_ showed slower rate of membrane binding, in agreement with what we previously observed for the kinetics of membrane fluidization experiments with Caco-2 cells (Figure 1c), but also slower membrane dissociation once the polymer is bound to the bilayer, *K*_a_ = 2.2 × 10^3^ M^–1^ s^–1^ and *K*_d_ = 3.3 × 10^–5^ s^–1^, respectively, compared with its cholesterol-terminated analogue Chol-(HEA)_70_ (*K*_a_ = 3.5 × 10^6^ M^–1^ s^–1^ and *K*_d_ = 3.1 × 10^–3^ s^–1^) (Table S3).

**Figure 3:**
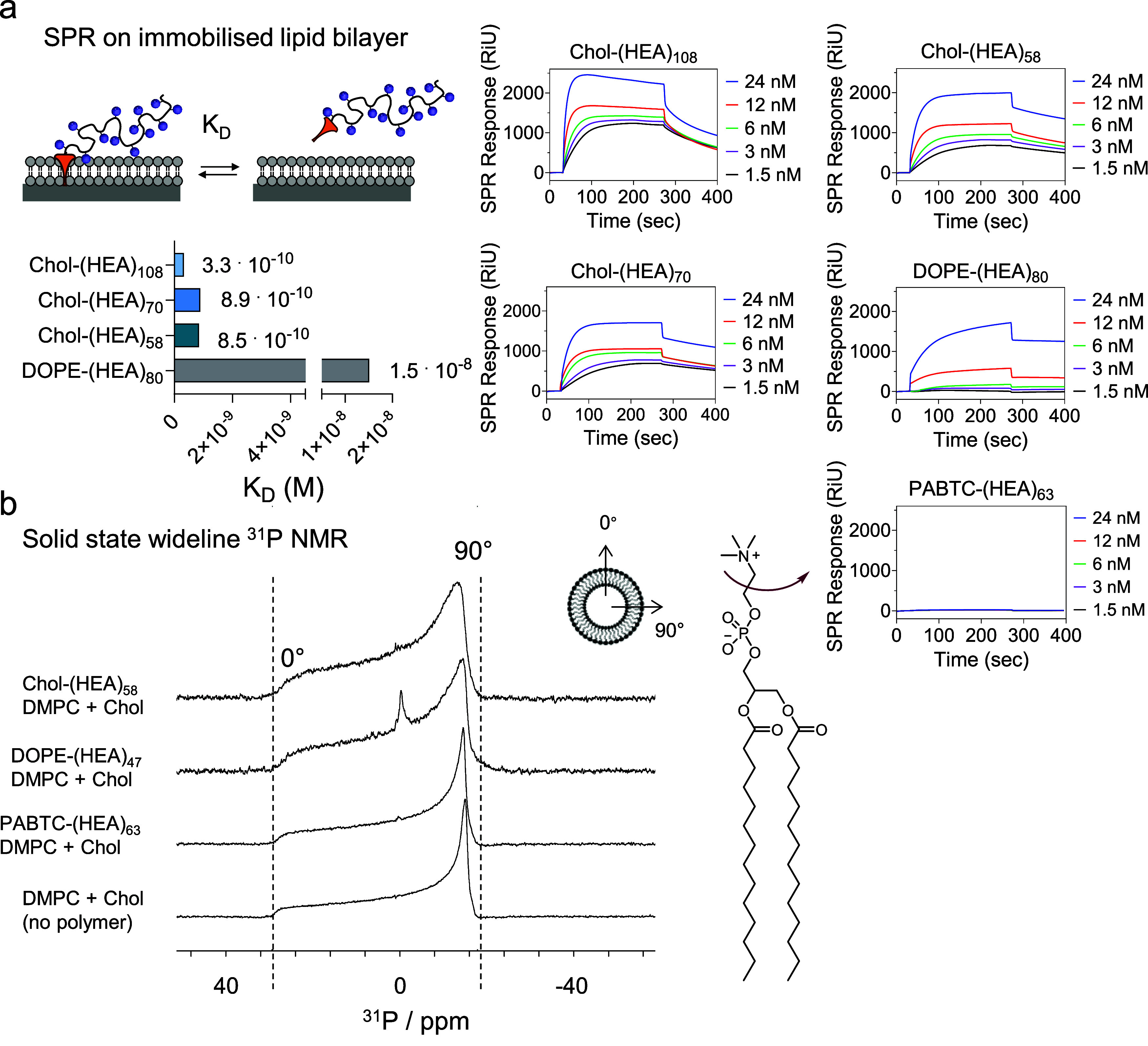
(a) Effect of polymer chain length and end-group on polymer binding to immobilized model lipid bilayer (DOPC:DSPC:cholesterol 2:1:1 molar ratio), as quantified by surface plasmon resonance (SPR). Left. The lipid bilayer was immobilized on an SPR L1 sensor chip, and the dissociation constant *K*_D_ of cholesterol- or DOPA-terminated poly­(2-hydroxyethyl acrylamide) (HEA)_n_ polymers was calculated. Higher *K*_D_ values are indicative of greater dissociation, hence weaker binding, of cholesterol- or DOPA-terminated (HEA)_n_ materials from the immobilized lipid bilayer. (b) Effect of cholesterol- or DOPA-terminated (HEA)_n_ materials on bilayer fluidity on model DMPC:cholesterol 1:1 membrane, as assessed by solid-state wide-line ^31^P NMR analysis. Polymers were tested at a membrane lipid:polymer 10:1 mol:mol ratio. The isotropic peak around 0 ppm in DOPE-(HEA)_47_ indicates the presence of nonbilayer structures, suggesting polymer-induced partial membrane shedding.

As expected, the control polymer PABTC-(HEA)_63_ showed no detectable interaction with the immobilized lipid bilayer, confirming again that the short butyl group on the polymer trithiocarbonate ω-group could not induce polymer insertion into the immobilized bilayer.

Next, the effect of membrane-inserting polymers on the fluidity of artificial membranes was assessed by solid-state wide-line ^31^P NMR analysis. This was done under the hypothesis that the interactions between Chol- and DOPE-(HEA)_n_ polymers and phospholipid membranes could alter the collective lipid dynamics within these bilayers, and that in turn this could affect their wide-line ^31^P NMR spectra. These spectra are dominated by effective chemical shift anisotropy (CSA) of 40–45 ppm, derived from the full ^31^P CSA of 200 ppm, partially averaged under fast axial rotation of the lipid molecules in membranes.

As for the previous ^13^C MAS ssNMR experiments, a simple model membrane formed by DMPC:cholesterol in a 1:1 molar ratio was utilized to facilitate the interpretation of spectra. Characteristic powder (Pake) patterns from the DMPC:cholesterol 1:1 membrane are shown in Figure 3B. The Pake distribution is affected by changes in molecular order, collective lipid motions, and dynamics that can arise from membrane perturbation by molecular additives.^61,62,71^ Specific changes in the powder distribution include (i) change in the overall width of the effective CSA pattern, reflective of changes in lipid order and dynamics, and (ii) deviation from the shape of the powder distribution, usually associated with increased bilayer flexibility, collective molecular librations, and membrane undulation.

Figure 3b shows ^31^P wide-line NMR of the lipid membrane incubated with Chol-(HEA)_58_, DOPE-(HEA)_47_ PABTC-(HEA)_63_ α-lipid polymers, possessing similar polymer chain lengths but different lipid end-groups. All ^31^P spectra are dominated by Pake intensity distributions, which confirms that the phospholipid DMPC remains in the lipid membrane phase in the presence of polymers (Figure 3b). The overall width of the powder pattern decreased slightly, from 44.4 to 41.4 ppm, in the presence of PABTC-(HEA)_63_, which revealed a very slight increase in lipid motions and chain order. However, no change in the shape of the distribution was detected, indicative of undisturbed molecular packing within the membrane. This indicates lack of PABTC-(HEA)_63_ polymer insertion into the membrane, which is in line with the observed very minimal change in membrane fluidity observed in the laurdan assay measurements (Figure 1a), as well as the absence of cross-polarization signals in the ^13^C MAS NMR spectrum (Figure 2b).

Conversely, the addition of DOPE-(HEA)_47_ to DMPC:cholesterol membranes led to pronounced changes in the ^31^P wide-line NMR spectrum. Besides a reduction in ^31^P effective CSA from 44.4 to 40.2 ppm, we observed a softening of the 0° and 90° edges of the distribution, which arises from an increased rate of angular lipid excursions (librations) and membrane undulation. This is also associated with an increase in lateral lipid diffusion along the curved surfaces of liposomes and associated angular excursions, which is indicative of enhanced membrane fluidity. This agrees with the results obtained in the laurdan assay, which showed that DOPE-(HEA)_47_ induces an increase in fluidity of the Caco-2 plasma membrane (Figure 1a). Incubation of the DMPC:cholesterol membrane with Chol-(HEA)_58_ also led to changes of the ^31^P wide-line NMR spectrum even more pronounced than those induced by DOPE-(HEA)_47_. Indeed, ^31^P CSA was reduced from 44.4 to 39 ppm and the 0° and 90° edges of the powder distribution were rounded even further (Figure 3b), revealing further enhancement in lipid angular and lateral excursions. Such changes are indicative of an increase in membrane fluidity induced by the insertion of Chol-(HEA)_58_ polymer into the DMPC:cholesterol membrane, again in agreement with the previous ^13^C CP MAS NMR (Figure 2b) and laurdan assay (Figure 1a) results.

Interestingly, the spectrum of DOPE-(HEA)_47_ showed an additional isotropic peak around 0 ppm (Figure 3b), suggestive of the presence of nonbilayer structures, in the form of smaller and faster tumbling DMPC aggregates shed from the main bilayer structure. When the same experiments were carried out with a more fluid model membrane containing only DMPC, this anisotropic signal was also present in the Chol-(HEA)_58_ sample, indicating that this effect is a function not only of the polymer chain-end but also of the composition of the lipid bilayer (Figure S23). This result agrees with recent studies by our group, where we observed a membrane composition-dependent decreased stability of bilayers, membrane shedding, and the coexistence of nonbilayer lipid environments and ordered membranes in the presence of relatively hydrophobic molecules such as butyl methacrylate^61^ and styrene.^62^

Taken together, this part of the study indicates that for the materials tested (i) α-cholesteryl (HEA)_n_ polymers have stronger association (lower *K*_D_) to DMPC membranes than analogous phospholipid-terminated DOPE-(HEA)_47_, (ii) binding kinetics for these materials were found to be significantly different, with phospholipid-terminated DOPE-(HEA)_80_ having slower association and dissociation rates than its cholesterol-terminated Chol-(HEA)_70_, and (iii) in line with the results of laurdan assay on Caco-2 cells, ^31^P wide-line NMR results showed that insertion of cholesteryl- and DOPE-terminated polymers increases the fluidity of model DMPC:cholesterol lipid membranes.

### Effect of Chol-(HEA)_n_ Polymers on Membrane Fluidity: Molecular Modeling

In parallel to the studies shown thus far, molecular modeling experiments were conducted to simulate the interactions between a simple dipalmitoylphosphatidylcholine (DPPC) bilayer membrane model and two model α-cholesteryl (HEA)_n_ homopolymers Chol-(HEA)_58_ and Chol-(HEA)_120_ with molar masses in the range of those utilized in our previous experiments. For atomistic modeling purposes, Chol-(HEA)_n_ polymers were divided into cholesterol, linker, HEA-derived repeating units (both enantiomers), and terminal units, with appropriate capping groups. Models for each were built using Chimera,^32^ and then unit parameter files were generated using AMBER’s Antechamber module.^33^ The models were relaxed using energy minimization and a short (5 ps) molecular dynamics (MD) simulation in implicit solvent, and then the final structures for the polymer were used to build a solvated system (TIP3P water model,^75^ 1 sodium counterion, and truncated octahedral unit cell with 63 Å cell vectors). After energy minimization and a 10 ns MD NPT equilibration, a 100 ns production simulation was run, saving coordinates every 100 ps. Data from this run was used to parametrize a 2-bead coarse grained (CG) model for HEA. The backbone was modeled as a P1 bead, and the side chain as a P2 bead.

In simulations of CG (HEA)_58_ in water, the polymer showed “molten globule” behavior, remaining relatively compact yet dynamic. RMSD from the initial structure showed a continuous increase, while the radius of gyration remained mostly constant at about 1.2 nm, with occasional peaks due to short-lived partial unfolding events. Compared with the initial structure, the mean distance matrix (i.e., mean of the distance matrices for each snapshot) showed little long-range order (Figure 4b).

**Figure 4:**
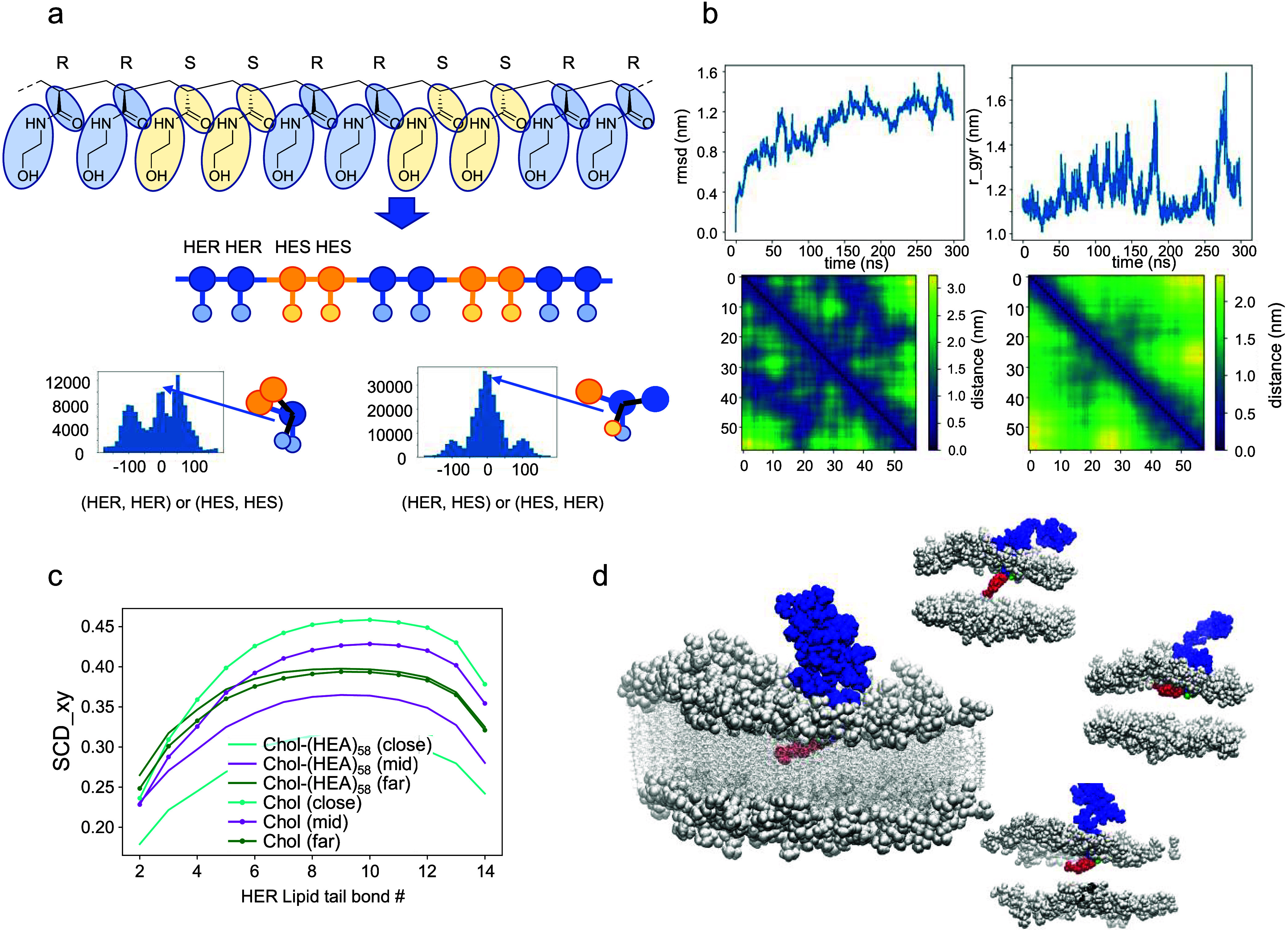
(a) Coarse-graining scheme for (HEA)_n_ polymers and details of polymer backbone dihedral distributions inferred from atomistic simulations. (b) Analysis of 300 ns CG simulations of atactic (HEA)_58_ in water. Top left: RMSD from initial structure; top right: radius of gyration; bottom left: distance matrix for initial structure; bottom right: mean distance matrix. (c) Deuterium order parameters calculated along lipid chains within radial shells: (a) within 2 nm, (b) between 2 and 5 nm, and (c) beyond 5 nm of the embedded cholesterol group. Data for control simulations (embedded cholesterol, no polymer) are also shown. (d) Snapshots from the 500 ns atomistic simulation of Chol-(HEA)_58_ in a DPPC bilayer. Blue: HEA, red: cholesterol: green: linker; gray: lipid with head groups shown space-filling and tails (left panel only) as rods.

Throughout the 500 ns production phase of the simulation, the cholesterol moiety remained firmly embedded in the lipid bilayer, while the -(HEA)_58_ chain retained a relatively compact globular conformation above the lipid surface (Figure 4d), in agreement with the observations from the CG simulations of (HEA)_58_. The system is quite fluid; however, the orientation of the cholesteryl polymer chain-end in the bilayer is quite variable, and at times the linker moiety and first few HEA units bury significantly into the bilayer surface (Figure 4d). This deviation from the canonical upright orientation of cholesterol within lipid bilayers, where its hydroxyl group is located near the lipid–water interface and its hydrocarbon tail is buried deep in the bilayer hydrocarbon region,^72^ is not unexpected, as the cholesteryl polymer chain-end does not possess a hydroxyl group, and by the motion of the hydrophilic (HEA)_58_ chain in the aqueous space above the bilayer is likely to induce a variable orientation of the cholesteryl polymer chain-end.

The disruption to the lipid packing resulting from this was evaluated by calculating the lipid deuterium order parameters (SCD_xy) along the lipid tails, separating the lipids into first the 20 closest to the embedded cholesterol group, then the next 30 closest, and finally all of the rest. The same analysis was performed for the control simulation, featuring a single cholesterol molecule within the DPPC bilayer. The results showed (Figure 4c) that on average those lipid chains closest to the cholesterol anchor in the Chol-(HEA)_58_ system were significantly less ordered than those further away, and that this effect was not seen for the control system (if anything, the cholesterol molecule alone seemed to increase lipid order, particularly toward the center of the bilayer). Using data from the last 300 ns of the Chol-(HEA)_58_ simulation, we calculated the mean membrane thickness (minimum distance between phosphorus atoms in the two leaflets) as a function of radial (*x*,*y*) distance from the inserted cholesterol, binning the data into radial slices of 0.5 nm width. We observed a small but significant decrease in membrane thickness (approximately 10%) within 2 nm of the insertion point (Figure S22).

The CG simulations of 16 independent Chol-(HEA)_n_ polymers absorbed onto a patch of DPPC membrane showed, individually, behavior that is fully consistent with the atomistic simulations. The cholesterol moieties remained firmly embedded within the bilayer, and the HEA “tails” retained their molten globule characteristics, appearing to be neither strongly attracted to nor repulsed from the membrane surface. There were, however, significant attractive interactions between HEA chains so that the anchored polymers diffused across the membrane surface from their initial positions to form aggregates (Figure 5). Polymer–polymer radial distribution functions showed a clear nearest-neighbor peak at 2.1 nm for the 58 mer, and 3.2 nm for the 120 mer. In the case of the 58 mer, comparing this value with the predicted radius of gyration of the polymer in aqueous solution (1.2 nm, see above), the aggregation was accompanied by slight compaction of the polymer structure. On going from the 58 mer to the 120 mer, we would expect the polymer volume to double, and so the polymer radius to increase by the cube root of this (1.26) if it was spherical. Since the ratio of the nearest-neighbor distances in the two aggregates is 1.52, this suggests that, when close to the membrane surface, the polymer globules are better regarded as oblate spheroids.

**Figure 5:**
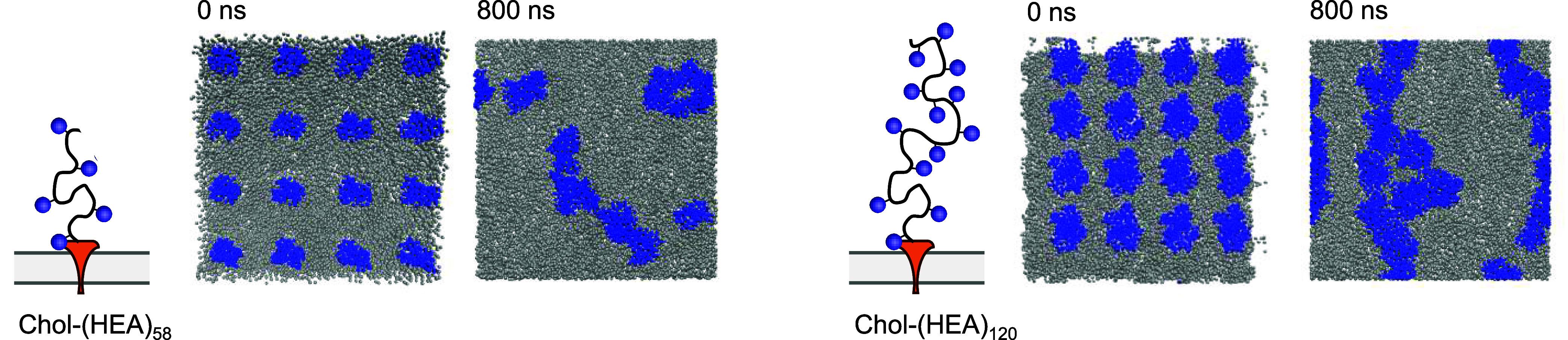
Aggregation simulations for Chol-(HEA)_58_ and Chol-(HEA)_120_ (blue) on a DPPC membrane surface (gray) (top view).

The simulations predict that Chol-(HEA)_n_ polymers firmly bind to the lipid membrane through their anchoring cholesterol groups. They predict that the (HEA)_n_ chain protrudes above the surface of the lipid bilayer and adopts a dynamic molten globule form and that the (HEA)_n_ chains are neither particularly attracted to nor repelled from the membrane surface. They also predict that close to the point of insertion of the cholesterol anchor into the membrane, there is significant disruption of the lipid organization, but that this effect decreases rapidly further away from this point. They predict that Chol-(HEA)_n_ polymers will tend to aggregate on the surface, but retain an oblate spheroid shape.

On the basis of these results, we can put forward a coherent hypothesis to explain the experimental observation that shorter Chol-(HEA)_n_ polymers induce greater membrane fluidity, as assessed by laurdan fluorescence, than longer ones. As the polymers self-assemble on the membrane surface, a “puddle” of the fluid membrane will form beneath each, close to the point of insertion of the cholesterol anchoring group. The longer the polymer chains, the larger the surface area each will occupy, and the wider spaced the points of insertion will be. As a result, on the macroscopic scale, the less fluid the membrane appears to be.

## Conclusions

Hydrophilic polymers bearing lipid functionalities at their α chain-end, defined here as α-lipid polymers, are successfully utilized for a range of applications spanning from the formulation of approved (bio)­therapeutics to the elucidation of biological processes. While existing studies used such materials to add specific functions to lipid formulations or living cells, e.g., to enhance the stability of nanoformulations and to add specific ligands to living cells, the effect of polymer insertion on the physicochemical properties of lipid membranes is still poorly understood. This study addresses this point by investigating polymer insertion from a lipid membrane perspective. To this aim, α-lipid polymers consisting of a hydrophilic chain and lipid anchors, cholesterol, and a model phospholipid (DOPE), analogous to those already present within cell plasma membranes, were ulilized.

Our data shows that, for the range of materials investigated, the nature of the membrane-inserting anchor, cholesterol vs DOPE phospholipid, influenced both insertion kinetics and extent of polymer insertion. Results show that, when compared at the same polymer chain length, cholesterol-terminated (HEA)_n_ materials lead to faster insertion into Caco-2 plasma membrane (Figure 1c) DOPC:DSPC:cholesterol 2:1:1 artificial membranes (Figure 3a and Table S3) and to the insertion of a larger number of polymer chains within Caco-2 cell membranes compared with their DOPE phospholipid-terminated counterparts.

Strikingly, data also show that the length of the hydrophilic polymer chains, rather than the chemical nature of their repeating units, plays a major role in the ability of the α-lipid polymers to insert within both artificial and plasma cell membranes. This was deduced in Caco-2 cells from our initial laurdan assay experiments (Figure 1a), and then specifically investigated using Chol-(HEA)_n_ α-lipid polymers. Within phospholipid bilayers, cholesterol increases the fluidity of phospholipid hydrocarbon chains below and decreases the fluidity above their lipid gel to liquid-crystalline phase transition temperature (*T*_m_), but under biologically relevant conditions, it tends to promote laterally more condensed and less permeable membranes.^73^ However, our cholesterol-terminated polymers lack the 3α-hydroxyl group which is believed to be critical for the ability of cholesterol to reduce the fluidity of lipid bilayers,^74^ thus we expect that our Chol-(HEA)_n_ α-lipid polymers would induce an increase in membrane fluidity.

Our results show that Chol-(HEA)_n_ materials with longer hydrophilic chains led a lower membrane fluidization effect on Caco-2 cell membranes (Figure 1a) and that this is likely due to the fact that with Chol-(HEA)_n_ with longer chains, a smaller number of polymer molecules can insert into cell plasma membrane, compared with their shorter counterparts, as observed by flow cytometry (Figure 2a). While this result is perhaps counterintuitive, one would expect that α-lipid polymers with larger hydrophilic polymer chains located in the extracellular space would result in a more significant disruption of the lipid bilayer; our data suggests that this effect is likely counterbalanced by that of a larger number of inserted shorter Chol-(HEA)_n_ polymer chains, which induce a more significant fluidization of cell plasma membranes. Molecular simulations further support this hypothesis, showing that for Chol-(HEA)_n_ polymers, a substantial disruption of the membrane lipid organization occurs near the point of insertion of the cholesterol anchor into the membrane, but that this effect declines further away from this point. Thus, for a given membrane area, a larger number of inserted polymer chains would induce a more pronounced increase in fluidity. Further work will be needed to investigate the additional effects of α-lipid polymer insertion on the physicochemical properties of lipid bilayers, for example, the effect on membrane bending rigidity. Indeed, although in this study the acyl chains within lipid bilayers appear to have enhanced disorder locally by insertion, how that translates to the global elastic properties of the membrane is currently not known.

Taken together, our experiments and modeling link the chemical structure of α-lipid polymers to changes in the physicochemical behavior of both artificial and cell lipid membranes. Thus, their implications are manifold: for some applications, changes of lipid organization and membrane, such as membrane fluidity, might not preclude their inclusion in specific biological systems or lipid formulations. However, the fluidity of plasma membranes does impact a number of cellular processes, including, cell signaling,^75^ gene expression in response to environmental changes,^76^ membrane protein function,^77^ and endocytosis.^78^ For liposomal formulations, membrane fluidity can affect their ability to interact with cells and pathogens and to overcome biological barriers.^24^ Thus, we postulate that the effect of polymer insertion on the physical and chemical properties of lipid bilayers will need to be considered when incorporating α-lipid synthetic polymers within effective formulations and biological systems.

## Supplementary Material



## Notes

Synthesis and characterization of α-lipid polymers and precursors, and MalPyr Blue dye (6). Description of cell culture, laurdan assay, flow cytometry experiments, SPR analysis, solid-state NMR experiments, and cytotoxicity assays (PDF)
