# Supplementary material for: Membrane-Inserting α‑Lipid Polymers: Understanding Lipid Membrane Insertion and Effect on Membrane Fluidity
Source: Chem Mater. 2025 Jul 18;37(15):5621–35. doi: 10.1021/acs.chemmater.5c00658 (PMC12355647; doi:10.1021/acs.chemmater.5c00658)
Supplement: Supplementary file 1 [file cm5c00658_si_001.pdf]

Supporting Information

Membrane-inserting α-lipid polymers: understanding lipid membrane insertion and effect on membrane fluidity.

Lorenzo Schiazza[a], Gokhan Yilmaz,[b] Pavel Gershkovich[a], Vivien Yeh,[c] Boyan Bonev,[c] Charles Laughton,[a,*] Snow Stolnik,[a,*] Giuseppe Mantovani,[a,*]

[a]School of Pharmacy, University of Nottingham, NG7 2RD, Nottingham, UK

[b]Department of Chemistry, University of Warwick, CV4 7AL, Coventry, UK.

[c]Biodiscovery Institute and School of Life Sciences, University of Nottingham, NG7 2RD, Nottingham, UK.

Materials and Methods

Reagents and cell culture media used for this study were purchased from Sigma-Aldrich (Dorset, UK), unless otherwise specified. PrestoBlue™ cell toxicity reagent, LDH cytotoxicity assay kit (Pierce™), laurdan dye (6-dodecanoyl-2-dimethylaminonaphthalene) and organic solvents were purchased from Fischer Scientific (Loughborough, UK). VA-044 was purchased from Wako Pure chemical Industries (Neuss, Germany). Phospholipids were purchased from Avanti Polar Lipids Inc (Alabaster, Alabama). Biacore Series S L1 chip was purchased from Cytiva Life Sciences (Sheffield, UK).

Human epithelial colorectal adenocarcinoma cells (Caco-2, American Type Culture Collection ATCC; Manassas, Virginia, passage 45-52) were used for this study.

Nuclear Magnetic Resonance (NMR) spectra were recorded on a Bruker-AV 400 at 400.13 MHz and referenced to solvent peaks. Coupling constants (J) are reported to the nearest 0.1 Hz, with peak multiplicities and assignments. Mestrenova 12 (Mestrelab Research, Santiago de Compostela, Spain) was used to process the spectra.

Polymer number-average molar mass ($M_{n,SEC}$), weight-average molar mass ($M_{w,SEC}$) and dispersity (Đ) were determined by Size Exclusion Chromatography (SEC), and calculated through Cirrus software and Microsoft Excel.

SEC was carried out using the following conditions, depending on polymer solubility:

i) Shimadzu GPC Prominence, LC-20AD (liquid chromatograph), SIL-20A HT (autosampler), RID 10A (Refractive index detector), CTO-20A (column oven). Tetrahydrofuran (THF), 2% Et_3N, 0.001% BHT as the mobile phase, two mixed-D columns connected in series, DI detector. Software LCsolution (Shimadzu). PMMA standards (Agilent EasyVials) were used for conventional calibration in the 500−955,550 g mol^{-1} range of molar masses. Number-average molar mass ($M_{n,SEC}$) and dispersity (Đ) values of synthesized polymers were determined by using Cirrus GPC software; ii) Agilent 1260 Infinity, detector WYATT technology DAWN 8+. Software OpenLAB and Excel. Mobile phase: HPLC water + 0.1 M $NaNO_3$; stationary phase: two Aquagel-OH columns connected in series, DI detector. PEO/PEG standards (Agilent EasyVials) were used for conventional calibration between 162−6,035,000 g mol^{-1}. Number-average molar mass ($M_{n,SEC}$) and dispersity (Đ) values of synthesized polymers were determined by using Agilent GPC software and Microsoft Excel; and iii) Polymer Laboratories PL-GPC 50, with PL-AS RT autosampler, *N,N*-dimethylformamide (DMF) + 0.1% LiBr as the mobile phase, mixed-D columns connected in series, DI detector. PMMA standards (Agilent EasyVials) were used for conventional calibration between 500−955,550 g mol^{-1}. Number-average molar mass ($M_{n,SEC}$) and dispersity (Đ) values of synthesized polymers were determined by using Cirrus GPC software.

RAFT agents.

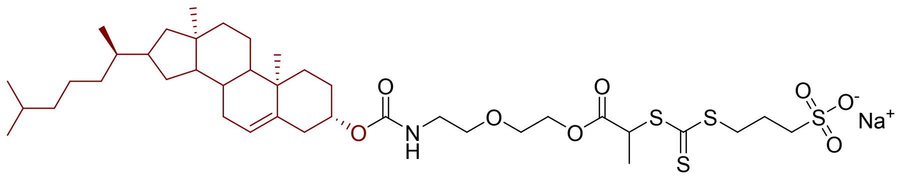

(1)

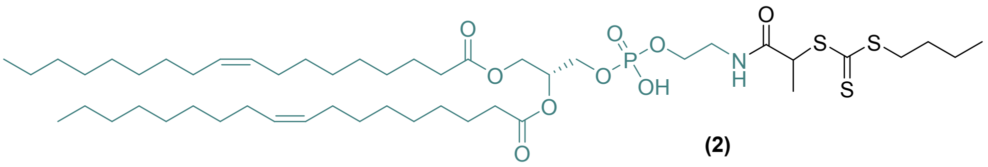

(2)

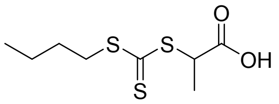

PABTC **(3)**

Chart S1. RAFT agents used in this work.

Cholesterol-based RAFT agent **(1)**[1], propanoic acid-2-[[(butylthio)thioxomethyl]thio]-carbonate (PABTC) RAFT agent [2], and PABTC-*O*-succinimide ester [2, 3] were synthesised as described previously.

Synthesis of DOPE RAFT agent (2)

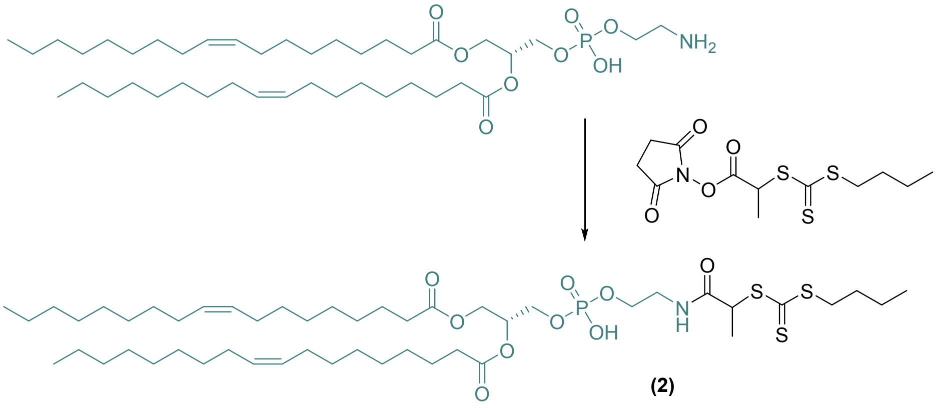

(2)

Scheme S1. Synthesis of 1,2-dioleoyl-sn-glycero-3-phosphoethanolamine (DOPE) RAFT agent **(2)**.

1,2-dioleoyl-sn-glycero-3-phosphoethanolamine (DOPE, 257 mg, 1 eq, 0.345 mmol) was solubilised in chloroform (15 mL) in a round-bottom flask and *N*-methylmorpholine was added (209 mg, 228 µL, 6.7 eq, 2.30 mmol). PABTC-*O*-succinimide **(2)**[2] (570 mg, 2.2 eq, 0.690 mmol) was solubilised in chloroform (25 mL) and added dropwise under stirring to the first solution, at room temperature. The reaction was left stirring for 7 days, progress was monitored by ^{1}H NMR. At completion the reaction mixture was washed with acidified (pH 3.9) water (3x40 mL). The solvent in organic phase was removed under reduced pressure to obtain DOPE RAFT agent **(2)** as a bright yellow, sticky oily residue (176 mg, 53% yield).

ESI-MS (expected m/z $[M]^-$ 962.55, found 962.54).

^{1}H NMR (400 MHz, DMSO-d6) δ 8.77 (t, *J* = 5.3 Hz, 1H, NH), 5.31 (t, *J* = 4.9 Hz, 4H), 5.08 (dq, *J* = 8.3, 4.8 Hz, 1H), 4.68 (dq, *J* = 14.6, 7.1 Hz, 2H), 4.27 (dd, J = 12.1, 3.1 Hz, 1H), 4.07 (dd, J = 12.0, 7.1 Hz, 1H), 3.72 (tq, *J* = 20.1, 6.1 Hz, 8H), 3.21 (q, J = 5.7 Hz, 2H), 2.34–2.17 (m, 4H), 1.97 (q, *J* = 6.3 Hz, 8H), 1.62 (pd, *J* = 7.4, 4.3 Hz, 4H), 1.49 (dd, *J* = 12.9, 7.2 Hz, 9H), 1.41–1.33 (m, 2H, CH_2), 1.32–1.15 (m, 37H), 0.92 – 0.78 (m, 12H).

^{13}C NMR (101 MHz, DMSO) δ 223.30, 172.94, 172.70, 172.03, 169.67, 130.07, 130.01, 70.81, 70.74, 64.54, 53.56, 50.48, 48.87, 43.92, 36.52, 34.03, 33.86, 31.77), 30.07, 29.61, 29.59, 29.33, 29.18, 29.13, 29.08, 29.02, 28.99, 28.93, 28.91, 27.09, 27.05, 24.94), 24.87, 22.58, 21.91, 17.38, 14.39, 13.89, 13.87.

FT-IR: 1750, 1680,1150, 1050 cm^{-1}.

ESI-MS (expected m/z $[M]^-$ 962.5443, found 962.5440

Synthesis of MalPyr Blue dye (6)

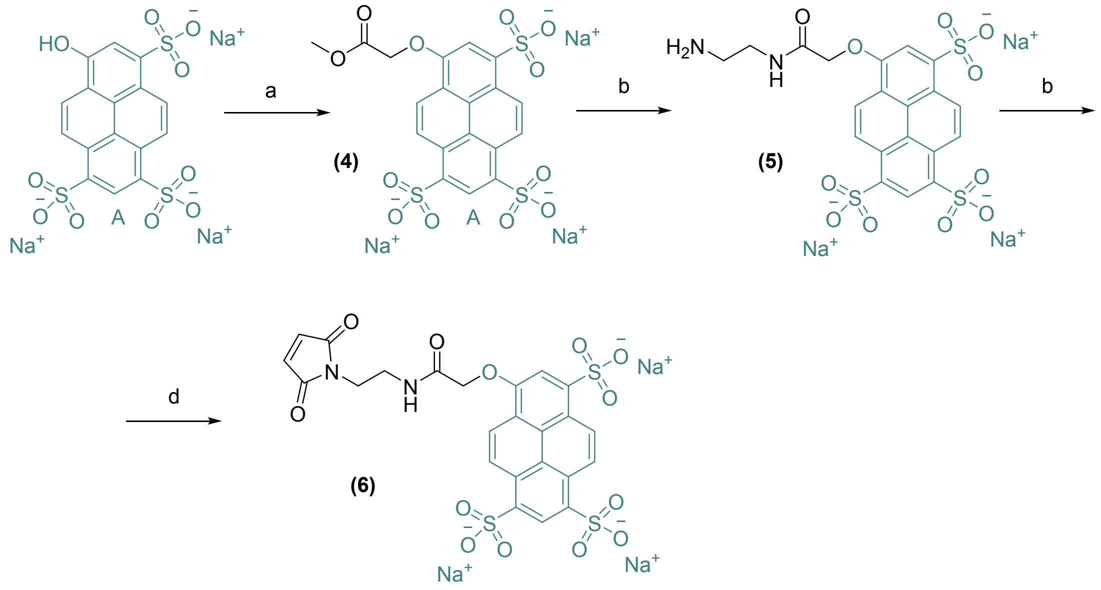

Scheme S2. Synthesis of MalPyr Blue dye. *Reagents and conditions*. a. 2-bromoacetic acid methyl ester, DIPEA, methanol reflux; b. ethylenediamine, methanol, room temperature; c. *N*-methoxycarbonylmaleimide, $NaHCO_3$ (aq), 0ºC to room temperature.

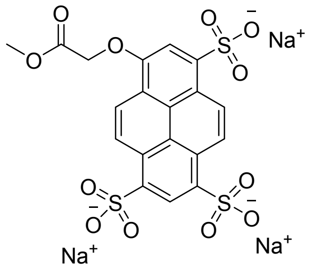

Pyranine methyl ester (4). Pyranine (1 eq, 3.09 g, 5.90 mmol) was solubilised in methanol (150 mL) under stirring in a round-bottom flask, protected from light, and the resulting solution was heated up to reflux. Methyl bromoacetate (3.8 eq, 3.41 g, 22.4 mmol) and *N,N*-diisopropylethylamine (DIPEA, 2.8 eq, 2.10 g, 16.5 mmol, 2.87 mL) were solubilised in methanol (30 mL) and added to the stirring reaction portion-wise (1/3 every 1.5 hours). After adding the last portion, the reaction was left refluxing for 3 additional hours. The solution was then cooled down to room temperature and transferred in a larger vessel, where isopropanol (50 mL) was added to precipitate the product. The precipitate was filtered and dried under reduced pressure, to give **(4)** as a dark yellow solid (1.61 g, 45% yield) which was used for the subsequent step without further purification.

^{1}H NMR (400 MHz, D_2O) δ 9.20 (s, 1H), 9.16 (d, *J* = 9.8 Hz, 1H), 9.09 (d, *J* = 9.7 Hz, 1H), 9.02 (d, *J* = 9.8 Hz, 1H), 8.92 (d, *J* = 9.6 Hz, 1H), 8.29 (s, 1H), 5.33 (s, 2H, CH_2), 3.91 (s, 3H, CH_3). ESI-MS (expected m/z $[M+H]^+$ 597.91 found 597.41

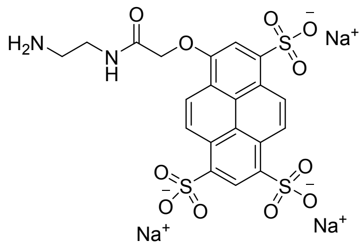

Pyranine ethylenediamine (5). Pyranine methyl ester **(4)** (1 eq, 1.61 g, 2.68 mmol) was solubilised in methanol (96 mL) in a round-bottom flask, protected from light. Ethylenediamine (2.5 eq, 0.403 g, 6.70 mmol, 0.450 mL) was added to this solution under stirring and the reaction was monitored by ^{1}H NMR by conversion of the 5.33 ppm CH_2 peak to 4.94 ppm. At completion, isopropanol (40 mL) was added to the reaction mixture until formation of a yellow precipitate **(5)**, which was filtered, and dried under reduced pressure, to obtain a bright yellow solid which was used to the following step without further purification (0.918 g, 57% yield).

^{1}H NMR (400 MHz, D_2O) δ 9.17 (s, 1H, aromatic CH), 9.08 (d, *J* = 9.8 Hz, 1H, aromatic CH), 9.02 (d, *J* = 9.7 Hz, 1H, aromatic CH), 8.95 (d, *J* = 9.9 Hz, 1H) , aromatic CH, 8.72 (d, *J* = 9.8 Hz, 1H, aromatic CH), 8.16 (s, 1H, aromatic CH), 4.94 (s, 2H, CH_2O), 3.54 – 3.48 (m, 1H, CH_2NH), 3.18 – 3.12 (m, 1H, CH_2NH), 2.88 (t, *J* = 6.2 Hz, 2H, CH_2NH_2), 2.83 (s, 1H, terminal amine). ESI-MS (expected m/z [M-3Na+2H] 557.00 found 557.00.

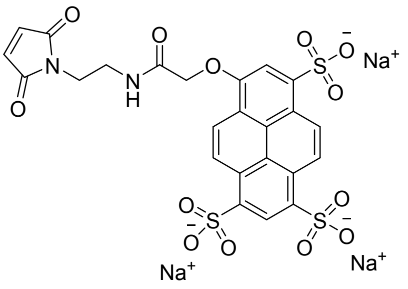

MalPyr Blue (6). Pyranine ethylenediamine **(5)** (1 eq, 0.800 g, 1.28 mmol) was solubilised in a saturated aqueous solution of $NaHCO_3$ (12.5 mL) in a round-bottom flask and the resulting solution was cooled to 0 °C. *N*-methoxycarbonylmaleimide (1 eq, 0.198 g, 1.28 mmol) was added and left stirring at 0 °C for 40 minutes, then at room temperature for further 24 hours. The reaction mixture was poured into a separating funnel containing brine:dichloromethane (40:30 mL), the organic phase (~ 40 mL) was collected and concentrated to 1/3 of the original volume under reduced pressure. The resulting solution was transferred to a larger vessel, where isopropanol (50 mL) was added to precipitate a yellow solid, that was collected via centrifugation. The precipitate was dried under reduced pressure, to give MalPyr Blue **(6)** as a yellow solid (950 mg, quant.).

^{1}H NMR (400 MHz, D_2O) δ 9.10 (s, 1H), 9.08 – 9.02 (m, 1H), 8.98 (d, *J* = 9.7 Hz, 1H), 8.91 (d, *J* = 9.9 Hz, 1H), 8.80 (d, *J* = 9.6 Hz, 1H), 8.18 (d, *J* = 3.2 Hz, 1H), 5.91 (s, 2H), 5.04 (s, 2H,

CH_2O), 3.44 (t, *J* = 5.5 Hz, 2H), 3.33 (t, *J* = 5.9 Hz, 2H). ESI-MS (expected m/z $[M-2Na+3H]^+$ 660.98, found 658.78).

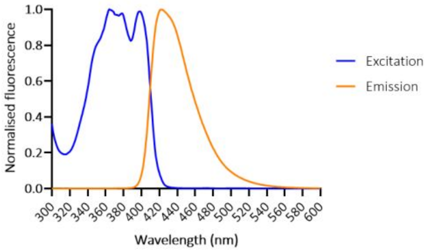

Figure S1. Normalised fluorescence spectrum of MalPyr Blue **(6)** in H_2O. UV-vis spectrum is also shown as a reference.

RAFT polymerisations. In a typical polymerisation reaction, the selected RAFT agent was solubilised in DMSO in a Schlenk tube, then the chosen monomer(s) was solubilised in deionised water (2 M monomer concentration) and added to the DMSO solution (DMSO:water 4:1 vol:vol). ^1H NMR analysis of an aliquot withdrawn from the reaction mixture before starting the polymerisation was carried out to confirm the exact initial RAFT agent:monomer(s) ratios. An appropriate volume of a 2.0 mg mL^{-1} aqueous stock solution of VA-044 radical initiator ([RAFT agent]:[VA-044] = 1:0.025 mol:mol) was added. The tube was then closed with a rubber seal, cooled to 0 °C, and deoxygenated by N_2 bubbling for 15 minutes. The Schlenk tube was then heated up to 70 °C, under vigorous stirring. After 2 hours, the reaction was stopped by rapid cooling (ice bath) and exposure to air. Finally, the reaction solution was analysed by ^1H NMR to determine the final monomer conversion. If the target monomer conversion (>90%) was not reached, another aliquot of VA-044 was added, the reaction mixture was cooled to 0 °C, deoxygenated by N_2 bubbling for 15 minutes, and finally heated at 70 °C under stirring for further 2 h.

To purify the polymers from unreacted monomers and reagents, the viscous reaction mixtures were diluted with CH_2Cl_2:MeOH 6:5 vol:vol, and precipitated (in acetone for Chol- and PABTC-terminated polymers, or tert-butyl methyl ether for DOPE- terminated polymers) in a falcon tube. The suspension was centrifuged (4500 rpm, 10 min), the supernatant was removed. The solid residue was dried under reduced pressure, then suspended in water, and purified by dialysis against 4L deionised water for 2-3 days, changing the solvent every 12-24 h. The dialysis was carried out using an appropriately sized MWCO membrane (1, 2, 5 or 7 kDa) and finally the polymer solutions were freeze-dried.

The degree of polymerisation (DP) of the isolated polymers was estimated by ^1H NMR, by comparing the integrals of specific polymer α-groups and those of the CH_2 and CH residues of the polymer backbone (1.6 and 2 ppm, respectively). TO estimate the DP of polymers synthesised with DOPE RAFT agent **(2)**, the signals for the DOPE CH_3 groups at 0.78 ppm

were used as internal reference. For polymers synthesised with cholesterol RAFT agent **(1)**, the signal used as reference was a cholesterol CH_3 triplet at 0.64 ppm. For the PABTC RAFT agent **(3)**, the signal used as reference was that of the CH_3 at 0.93 ppm.

Table S1. Characterisation of polymers synthesised in this study.

Code	DP[a]	HEA [b]	AAPA[b]	AA[b]	$M_{n,NMR}$ (kDa)[a]	*Đ*[c]
Chol-$(HEA)_{52}$	52	52	-	-	7.0	1.14
Chol-$(HEA)_{70}$	70	70	-	-	9.1	1.08
Chol-$(HEA)_{108}$	108	108	-	-	13.5	1.07
Chol-$(AA)_{54}$	54	-	-	54	4.9	1.13
Chol-$(AA)_{84}$	84	-	-	84	7.1	1.12
Chol-$[(HEA)_{0.9}$-*r*-$(AAPA)_{0.1}]_{54}$	54	48	6	-	7.4	1.23
Chol-$[(HEA)_{0.9}$-*r*-$(AAPA)_{0.1}]_{140}$	140	126	14	-	17.6	1.10
Chol-$[(HEA)_{0.9}$-*r*-$(AA)_{0.1}]_{51}$	51	46	-	5	6.7	1.19
Chol-$[(HEA)_{0.9}$-*r*-$(AA)_{0.1}]_{57}$	57	49	-	8	7.3	1.06
Chol-$[(HEA)_{0.9}$-*r*-$(AA)_{0.1}]_{105}$	105	94	-	11	12.7	1.20
Chol-$[(HEA)_{0.7}$-*r*-$(AA)_{0.3}]_{52}$	52	37	-	15	6.4	1.18
Chol-$[(HEA)_{0.7}$-*r*-$(AA)_{0.3}]_{94}$	94	75	-	19	11.1	1.18
PABTC-$(HEA)_{50}$	50	50	-	-	6.0	1.29
PABTC-$(HEA)_{63}$	63	63	-	-	7.5	1.06
PABTC-$(HEA)_{80}$	80	80	-	-	9.5	1.24
PABTC-$(AA)_{37}$	37	-	-	37	2.1	1.05
PABTC-$(AA)_{140}$	140	-	-	140	10.4	1.23
PABTC-$[(HEA)_{0.9}$-*r*-$(AAPA)_{0.1}]_{46}$	46	39	7	-	5.7	1.15
PABTC-$[(HEA)_{0.9}$-*r*-$(AAPA)_{0.1}]_{81}$	81	73	8	-	9.8	1.16
PABTC-$[(HEA)_{0.9}$-*r*-$(AA)_{0.1}]_{45}$	45	40	-	5	5.2	1.11
PABTC-$[(HEA)_{0.9}$-*r*-$(AA)_{0.1}]_{75}$	75	70	-	5	8.6	1.15
PABTC-$[(HEA)_{0.7}$-*r*-$(AA)_{0.3}]_{45}$	45	40	-	5	5.2	1.11
PABTC-$[(HEA)_{0.7}$-*r*-$(AA)_{0.3}]_{80}$	80	67	-	13	8.9	1.28
DOPE-$(HEA)_{47}$	47	47	-	-	6.4	1.15
DOPE-$(HEA)_{80}$	80	80	-	-	10.2	1.11
DOPE-$(AA)_{42}$	42	-	-	42	4.0	1.04

[a] Degree of polymerisation (DP), calculated by ^1H NMR as described in the *RAFT polymerisations* section above). [b] HEA = hydroxyethyl acrylamide, AAPA 3-acrylamido propanoic acid, AA = acrylic acid. [c] Molar mass dispersity (M_w/M_n, *Đ*) was determined by SEC as described in the *materials and methods* section above.

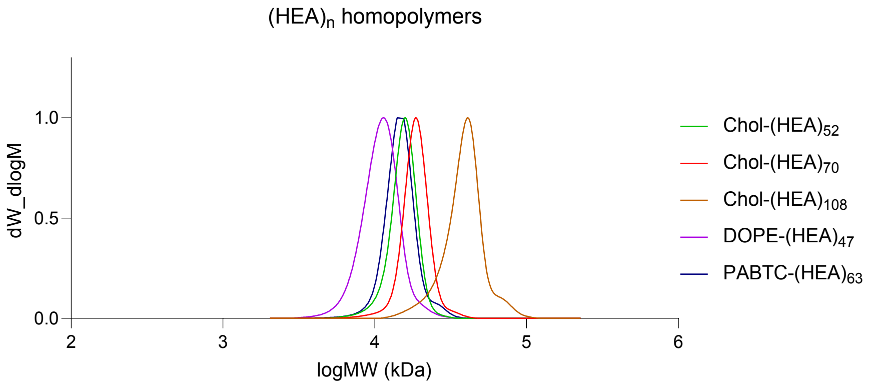

Figure S2. Normalised SEC traces of $(HEA)_n$ homopolymers. Analyses were performed using DMF + 0.1 % LiBr as the mobile phase (PMMA standards).

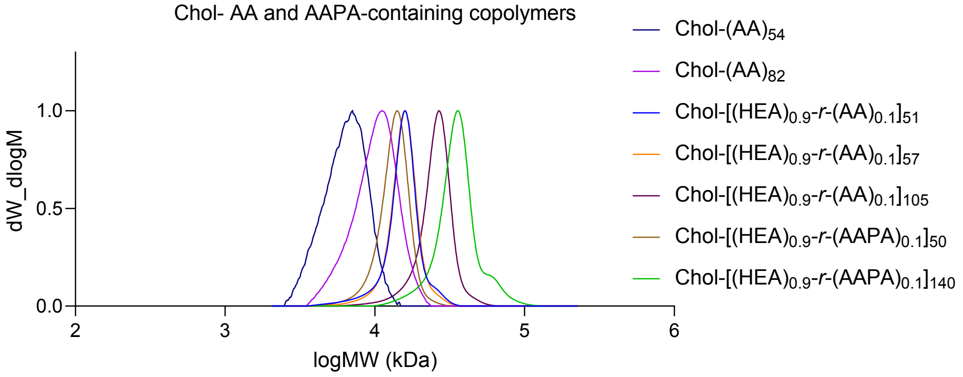

Figure S3. Normalised SEC traces of HEA, AA and 3-AAPA (co)polymers. Analyses were performed using DMF + 0.1 % LiBr as the mobile phase (PMMA standards).

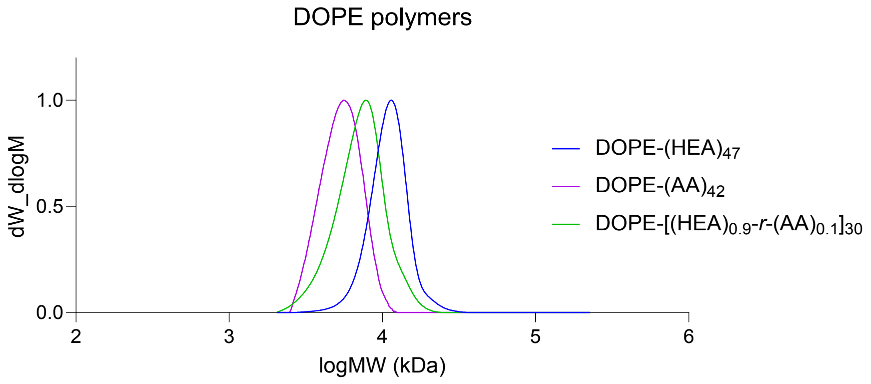

Figure S4. Normalised SEC traces of DOPE HEA, and AA (co)polymers. Analyses were performed using DMF + 0.1 % LiBr as the mobile phase (PMMA standards).

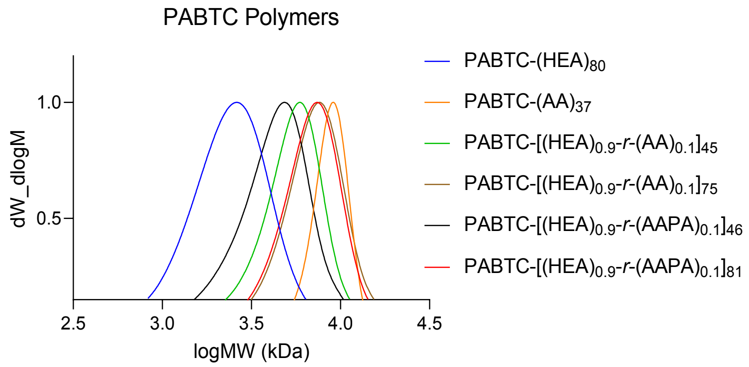

Figure S5. Normalised SEC traces of PABTC HEA, AA and 3-AAPA (co)polymers. Analyses were performed in HPLC water + 0.1 M $NaNO_3$ (PEO/PEG standards).

Tagging of $(HEA)_n$ polymers with MalPyr Blue

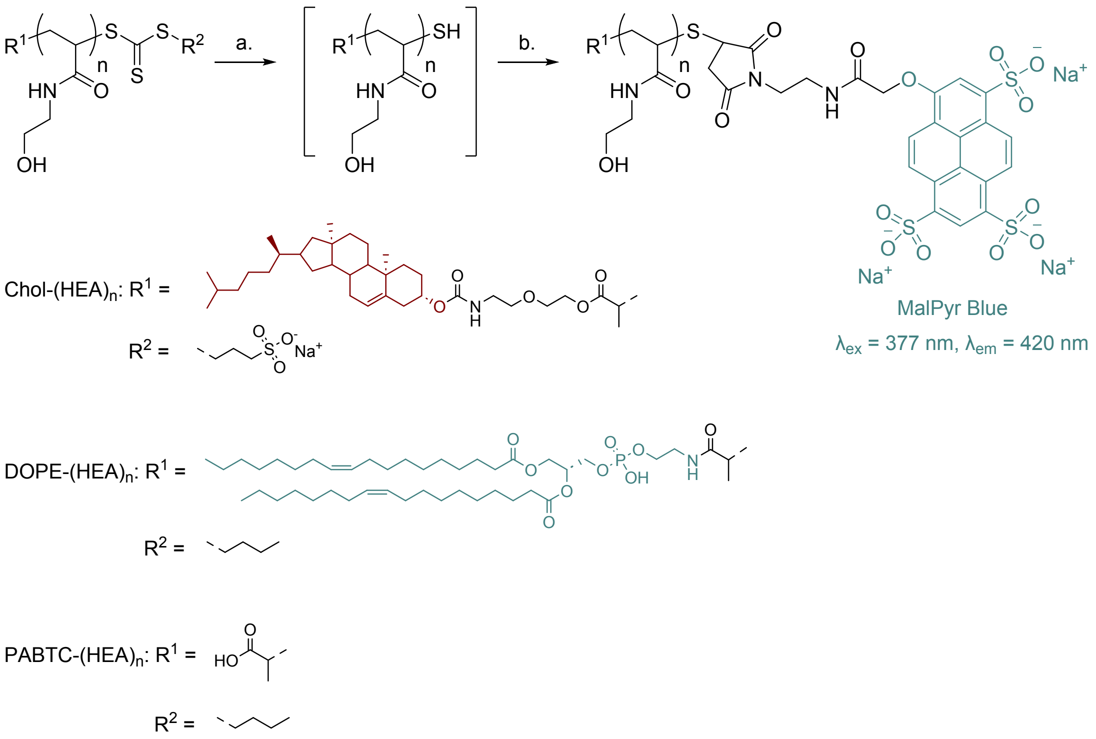

Scheme S3. Synthesis of MalPyr Blue fluorescently-tagged $(HEA)_n$ polymers. *Reagents and conditions*: a. Benzylamine, TCEP, H_2O, 1 h, room temperature; b) MalPyr Blue **(6)**, H_2O, 10 h, room temperature, dark.

The selected polymer (1 eq) was solubilised in water (800 µL per 100 mg of polymer) in a round-bottom flask. The flask was sealed, and the solution degassed for 15 minutes by argon bubbling. Benzylamine (10 eq) and tris(2-carboxyethyl)phosphine (TCEP 0.01 eq) were added and the reaction solution was stirred for 1 hour (or until the solution went from yellow to colourless), and room temperature. MalPyr Blue **(6)** (8 eq) was then added, and the resulting solution was stirred protected from light. In both reaction steps the reaction solution was degassed by argon bubbling after each addition. After 10 hours, isopropanol (20 mL) was added to precipitate the polymer, and the excess of unreacted starting materials was removed by dialysis against deionised water (2 days, with several water changes, MWCO 1 kDa). Following freeze-drying, the polymers were isolated as pale-yellow solids.

Successful conjugation was confirmed through SEC-HPLC. Analysis of fluorescently tagged polymers was performed with a Shimadzu UFLC HPLC with a SPD-M20A Diode array detector (Deuterium and Wolframium) and a RF-10XL fluorescence detector (Xenon lamp). A SEC Aquagel-OH column was used as stationary phase, while the mobile phase was HPLC-grade water. The fluorescence detector was set at λ_{ex} = 377 nm and λ_{em} = 420 nm.

Estimation of tagging efficiencies, i.e. the percentage of polymer chains tagged with MalPyr Blue, was carried out on 0.05 and 0.1 µM HBSS solutions of the fluorescently tagged polymers and of MalPyr Blue **(6)** free dye. The solutions were transferred in a 96-well plate (Costar black bottom), 150 µL per well. The fluorescence intensity measurement was run on a Spark 10M Plate Reader (Tecan). The analysis was performed at 37 °C, with λ_{ex} = 377 nm and λ_{em} = 420 nm. Characterisation of MalPyr Blue-tagged $(HEA)_n$ is shown on Table S2.

Table S2. Characterisation MalPyr Blue fluorescently tagged $(HEA)_n$ polymers.

Code	Tagging efficiency (%)[a]	$M_{n,NMR}$ (kDa)[b]	$Đ$[c]
MalPyr Blue Chol-$(HEA)_{52}$	83±7	7.5	1.07
MalPyr Blue Chol-[$(HEA)_{70}$	105±10	9.7	1.21
MalPyr Blue Chol-[$(HEA)_{108}$	113±3	14.0	1.12
MalPyr Blue DOPE-$(HEA)_{47}$	91±4	7.0	1.10
MalPyr Blue PABTC-$(HEA)_{50}$	102±2	6.6	1.11

[a] Tagging efficiency, calculated by comparing the fluorescence emission intensity of the MalPyr Blue dye and the MalPyr Blue-tagged polymers (λ_{ex} = 377 nm and λ_{em} = 420 nm), at the same molar concentration (0.05 and 0.1 µM in HBSS). Tagging efficiencies shown in the table are the averaged values between the two concentrations (n=12). [b] Number-average molecular weight M_n was calculated by ^{1}NMR as previously described. [c] Molar mass dispersity (M_w/M_n, $Đ$) was determined by SEC using DMF + 0.1 LiBr as the mobile phase. The system was calibrated using PMMA standards (500−955,550 g mol^{-1}).

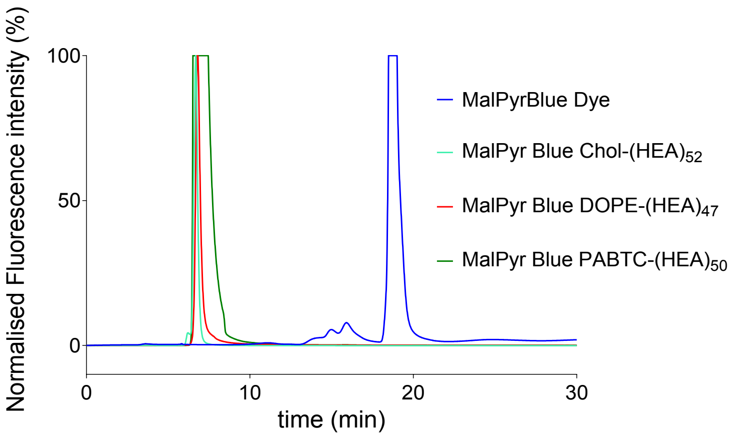

Figure S6. SEC-HPLC chromatograms with fluorescence detection (λ_{ex} = 377 nm and λ_{em} = 420) for MalPyr Blue **(6)**-tagged Chol-(HEA)$_n$ polymers, confirming conjugation of MalPyr Blue **(6)** to Chol-(HEA)$_n$ polymers.

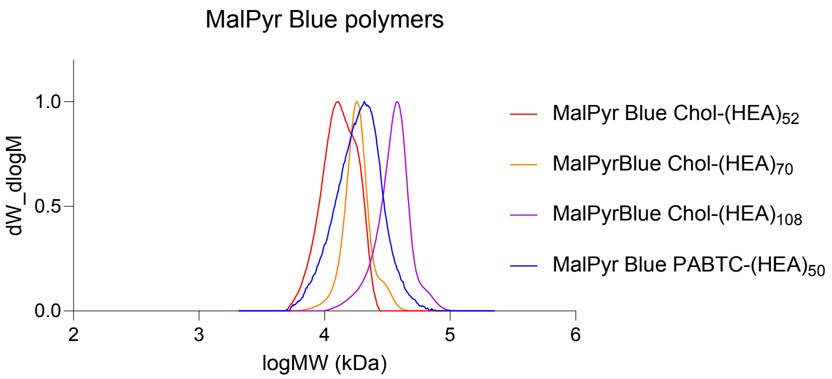

Figure S7. Normalised SEC traces of MalPyr Blue **(6)**-tagged Chol-(HEA)$_n$ polymers. Analyses were performed using DMF + 0.1 % LiBr as the mobile phase (PMMA standards).

Critical aggregation concentration (CAC) of the polymers synthesised in this work.

The estimation of the CAC of the polymers synthesised in this work was carried out using the pyrene method.[4] A saturated pyrene solution was prepared by first dissolving 5 mg of pyrene in 10 mL methanol and then diluted 1:20 in methanol. An aliquot (50 µL) of this solution was mixed with 3 mL of a 10 mg/mL polymer solutions in HPLC-grade water. The fluorescence emission at λ_{em} = 373 nm and λ_{em} = 385 nm of the prepared pyrene-polymer solutions was measured, with a λ_{ex} = 340 nm. The solutions were subjected to serial dilutions, using the saturated pyrene suspension, and subject to further fluorescence emission measurements.

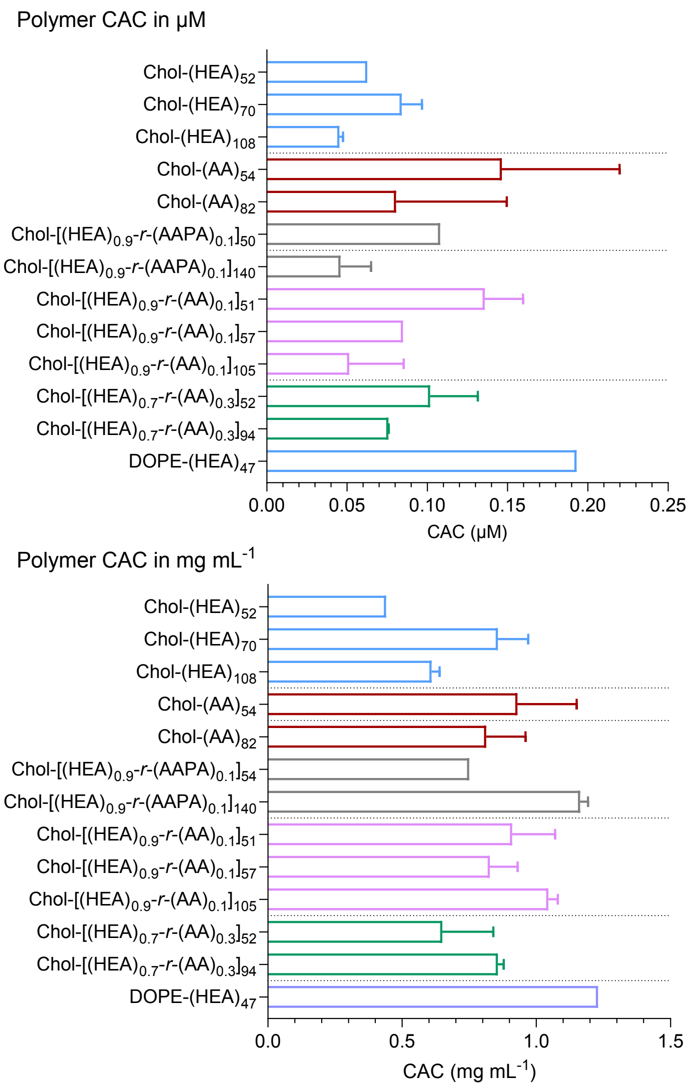

Figure S8. Critical aggregation concentration (CAC) for selected homo and copolymers synthesised in this study as determined using pyrene as the fluorescence probe, and expressed in μM (top) and mg mL^{-1} (bottom) (n = 1-3, SD).

2.7 Laurdan assay

Caco-2 cells were seeded at concentration $1*10^4$ per well in 96-well plates (black) and cultured for 24 hours. The medium was removed from cells and these were incubated with a 2 μM solution of laurdan dye in HBSS, for 30 minutes[5]. Following exposure, the dye was washed off twice with PBS, then cholesterol-, DOPE-, and non membrane-inserting PABTC-terminated control polymers at the chosen concentration(s) in HBSS (150 μL) were added to the wells. After 30 minutes of incubation, fluorescence was measured at 360/460 nm ($\lambda_{ex}/\lambda_{em}$) and at 360/490 nm ($\lambda_{ex}/\lambda_{em}$), at 37 °C.

Generalised Polarisation (GP) of Laurdan was calculated by subtracting readings for the blank samples (Caco-2 cells + medium, to account for cell auto fluorescence) to the values

measured for each well containing cells and Laurdan, using the following formula (Equation S1):

$$GP = \frac{(I_{460} - I_{490})}{(I_{460} + I_{490})}$$

Equation S1. Generalised Polarisation (GP) of Laurdan. I_{460} and I_{500} represent fluorescence emission intensities at λ_{em} = 460 nm and λ_{em} = 490 nm respectively, following excitation at λ_{ex} = 360 nm.

The 96-well plates were analysed with a Spark 10M Plate Reader and data was recorded using a SparkControl 2.1 software. Data was processed with Prism 9.4.1 (GraphPad).

To test whether the presence of membrane-inserting polymers in the cell medium affected the fluorescence readout in the laurdan assay, following cell incubation with Chol-$(HEA)_{58}$ and PABTC-$(HEA)_{63}$ (control polymer) in HBSS (150 µL) and reading of fluorescence at 360/460 nm ($\lambda_{ex}/\lambda_{em}$) and at 360/490 nm ($\lambda_{ex}/\lambda_{em}$), the polymer-containing supernatant was removed and transferred it in a second 96-well plate, and replaced with fresh HBSS.

Fluorescence emission for both 96-well plates was recorded to:

i) observe whether a proportion of Laurdan was removed when the polymer-containing supernatant was removed;

ii) confirm that the membrane fluidity change, expressed as ΔGP, was still present on cells after removing any potential effect from interaction between the polymers and laurdan dye molecules

Polymer concentrations and calculated ΔGP values are shown in Figure S9 below.

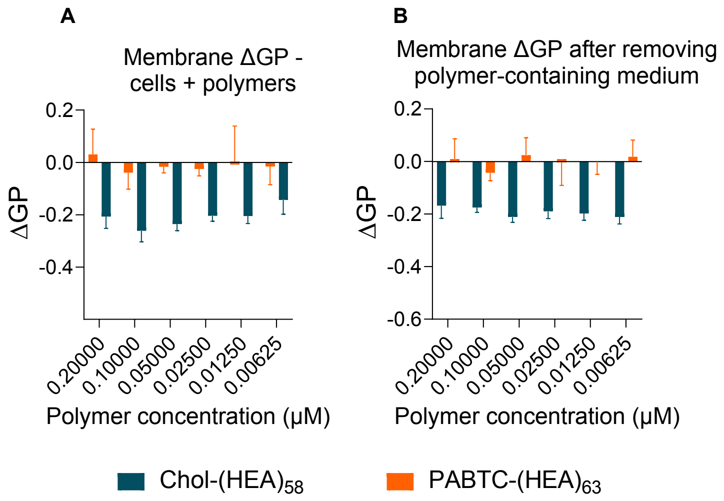

Figure S9. Effect of the presence of membrane-inserting polymer Chol-$(HEA)_{58}$ and control polymer PABTC-$(HEA)_{58}$ in the cell medium on the ΔGP on laurdan-treated Caco-2 cells. **A** ΔGP values obtained for Caco-2 cells treated with Laurdan, in a polymer-containing cell medium. **B** ΔGP values of Caco-2 cells after medium at point A was replaced with fresh, polymer-free, cell medium. (n = 6, error bars expressed as SD). This control experiments confirmed that, as expected, the ΔGP observed in the laurdan assay experiments was due to the fluidization of cell plasma membrane induced by polymer insertion.

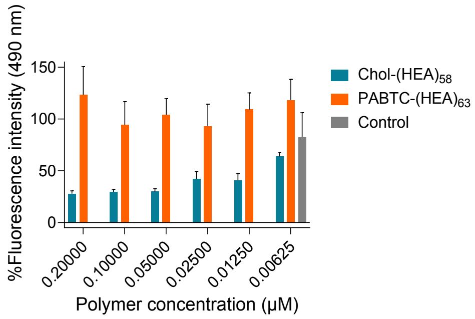

Figure S10. Effect of the presence of membrane-inserting Chol-$(HEA)_{58}$ and control PABTC-$(HEA)_{63}$ polymer in the cell medium on the laurdan fluorescence emission of laurdan-treated Caco-2 cells. Following incubation with polymers at various concentrations for 30 min, the supernatant was removed and replaced with fresh cell medium. Fluorescence intensity (%) of (λ_{ex} = 360 nm; λ_{em} = 440 and 490 nm) of laurdan-treated cells (top) and polymer-containing supernatant are calculated on a well-to-well basis, using the starting fluorescence intensity (cells+bound Laurdan+polymers) for each well as 100% (n = 6, error bars expressed as SD).

Flow Cytometry of Caco-2 cells treated with MalPyr Blue-tagged $(HEA)_n$ polymers.

Caco-2 cells were seeded at concentration 1*10^4/mL in 12-well plates in phenol-red free EMEM. After 24 hours incubation, the medium was removed from cells, and these were treated with an appropriate volume (1 mL) of the fluorescently tagged polymer solutions (0.1 µM in HBSS) for a 20-minute incubation. The fluorescent polymer solutions were removed, and cells were washed twice with PBS to remove the polymer solutions. The cells were exposed to an appropriate quantity (0.3 mL per well) of a pre-warmed (37 °C) trypsin-EDTA solution. Cells were incubated for 5 minutes in the standard cell culture conditions. Then, an equal amount (0.3 mL) of pre-warmed phenol red-free medium was added to the detached cells, to inhibit further effect of trypsin and to recover as many cells as possible. The cell suspensions were transferred into 1.5 mL Eppendorf vials for centrifugation (250 g for 4 minutes), to obtain a pellet of cells. The supernatant was aspirated, and the cell pellet was suspended in 40 µL of room temperature PBS. Cells were stored on ice and covered from light for 2 hours, and then were analysed on Amnis® ImagestreamX® MKII Imaging Flow Cytometer (Luminex).

The images and data were elaborated using the IDEAS® 6.0 software (LuminexCorp). The results are expressed as adjusted Median Fluorescence Intensity (aMFI), calculated as:

aMFI=(median fluorescence intensity (sample))/(median fluorescence intensity (untreated cells)×Tagging efficiency).

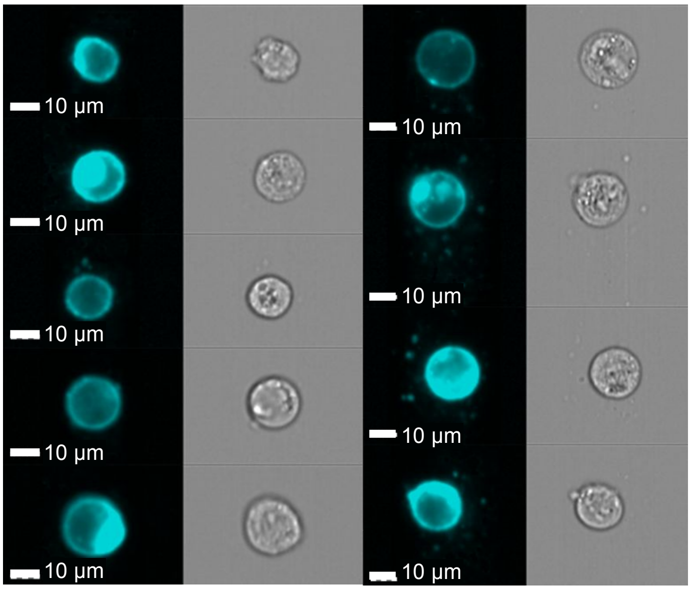

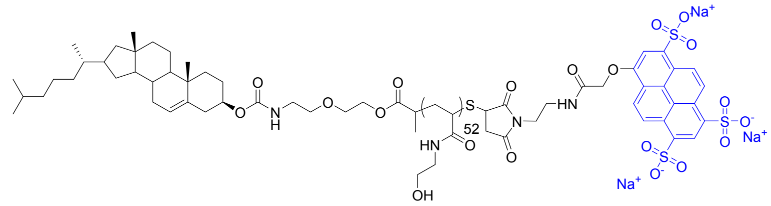

MalPyr Blue Chol-$(HEA)_{52}$

Figure S11. Flow cytometry images for Caco-2 cells treated with MalPyr Blue Chol-$(HEA)_{50}$, showing fluorescence (λ_{ex} = 377 nm and λ_{em} = 420 nm) and brightfield images.

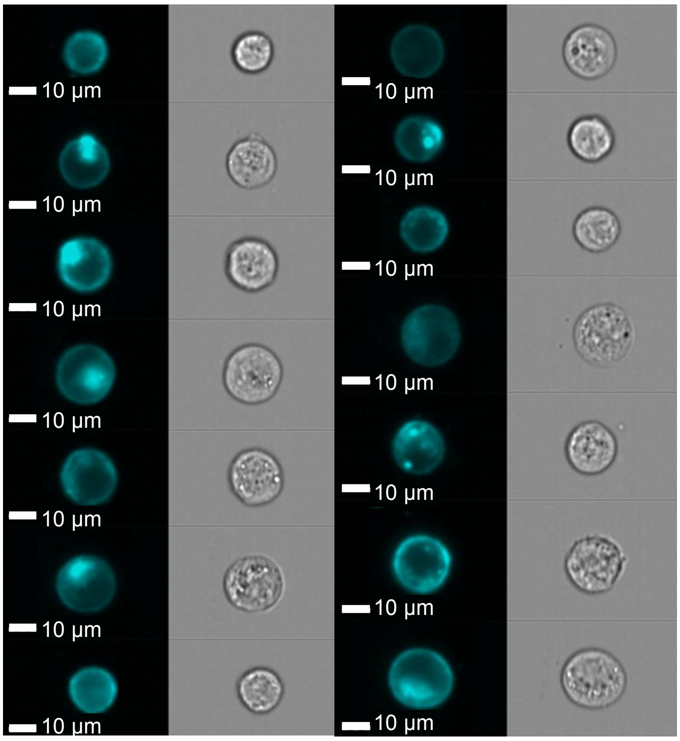

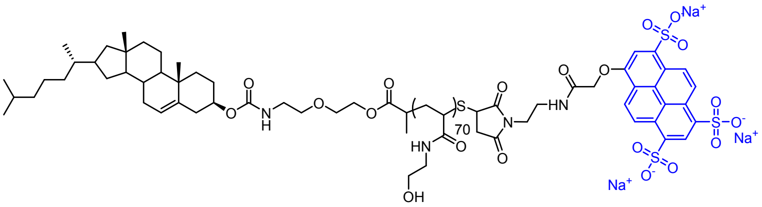

Chol-(HEA)$_{70}$

Figure S12. Flow cytometry images for Caco-2 cells treated with MalPyr Blue Chol-(HEA)$_{70}$, showing fluorescence (λ_{exc} = 377 nm and λ_{em} = 420 nm) and brightfield images.

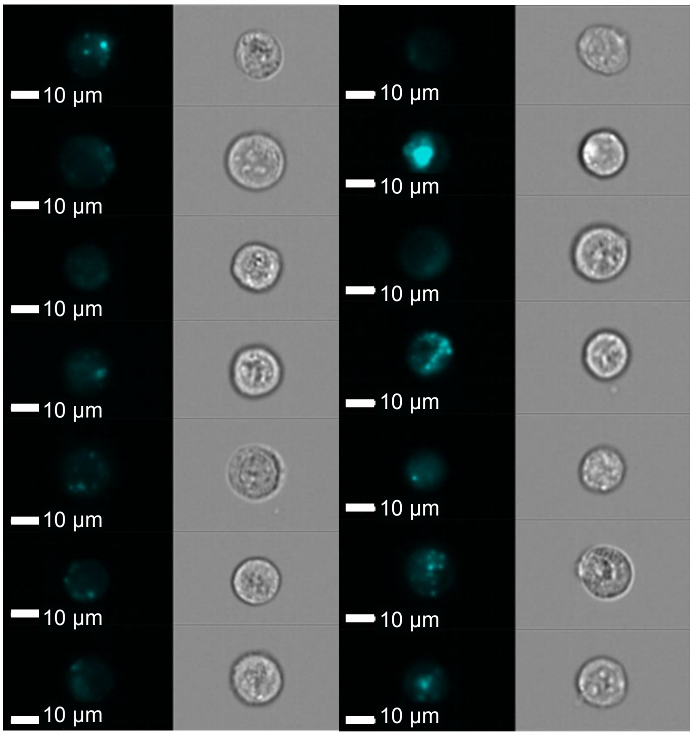

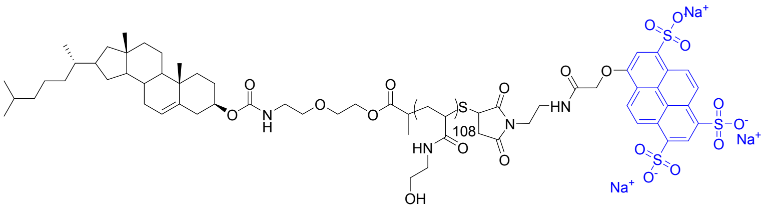

MalPyr Blue Chol-(HEA)$_{108}$

Figure S13. Flow cytometry images for Caco-2 cells treated with MalPyr Blue Chol-(HEA)$_{108}$, showing fluorescence (λ_{ex} = 377 nm and λ_{em} = 420 nm) and brightfield images.

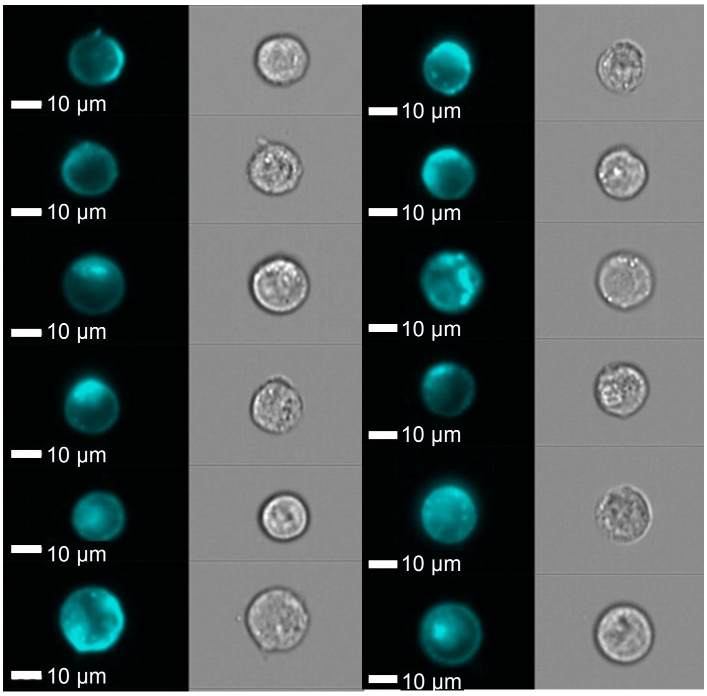

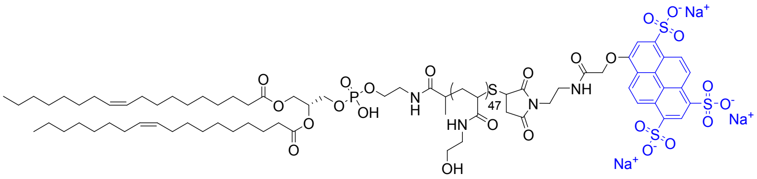

MalPyr Blue DOPE-(HEA)$_{47}$

Figure S14. Flow cytometry images for Caco-2 cells treated with MalPyr Blue DOPE-(HEA)$_{47}$, showing fluorescence (λ_{ex} = 377 nm and λ_{em} = 420 nm) and brightfield images.

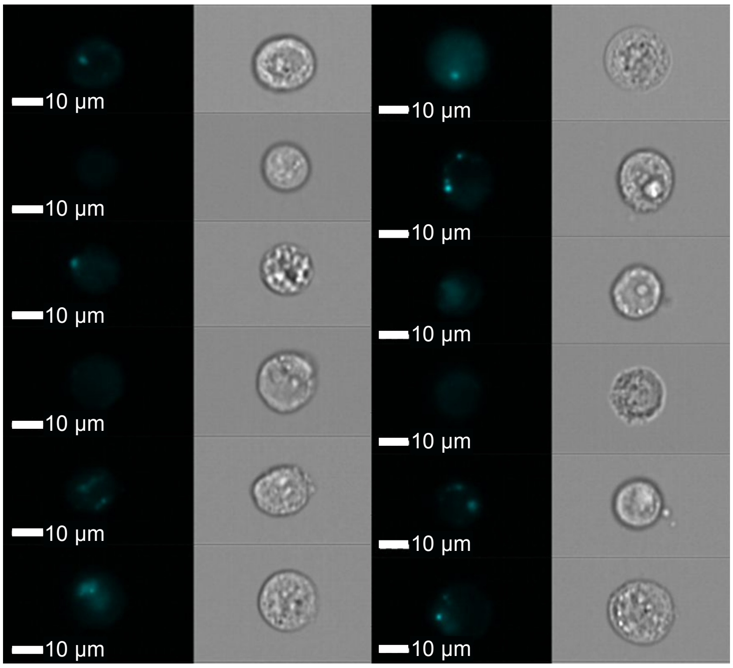

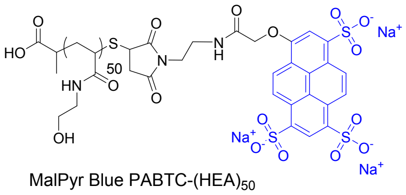

MalPyr Blue PABTC-(HEA)$_{50}$

Figure S15. Flow cytometry images for Caco-2 cells treated with MalPyr Blue PABTC-(HEA)$_{50}$, showing fluorescence (λ_{ex} = 377 nm and λ_{em} = 420 nm) and brightfield images.

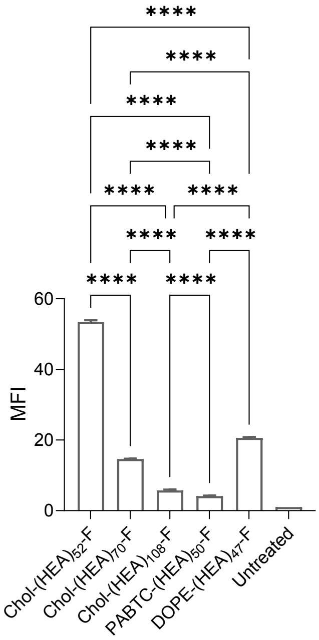

Figure S16. Insertion of cholesterol- and DOPE-teminated (HEA)$_n$ polymers in the plasma membrane of Caco-2 cells depends on polymer chain length and nature of polymer chain-end. Caco-2 cells were

incubated for 20 min with 0.1 μM polymer solutions and analysed by imaging flow cytometry. Statistical significance was calculated with one-way ANOVA. Error bars indicate s.d. (**** P<0.0001).

Surface Plasmon Resonance (SPR) analysis

The interaction between cholesterol-, phospholipid DOPE, and non membrane-inserting PABTC-terminated polymers and immobilised lipid bilayers was performed on a BIAcore T200 system (GE Healthcare). The SPR measurements were carried out after depositing an immobilised lipid bilayer by passing liposomes on a BiaCore L1 chip (Cytiva Life Sciences, Sheffield, UK).

Solutions of selected polymers were prepared at different concentrations (24 to 1.5 nM) in HBSS buffer solution. Sensorgrams for each polymer concentration were recorded with a 240 sec injection of polymer solution (ON period) followed by 120 sec of buffer alone (OFF period). Kinetic data was processed using a single set of sites (1:1 Langmuir binding) model in the BIAevalulation 3.1 software.

Liposome preparation. Liposomes were prepared following gentle hydration method[6, 7], followed by membrane extrusion[8]. Briefly, phospholipid stock solutions (2.5 mg/mL in CH_2Cl_2) were used to solubilise solid cholesterol in a round-bottomed flask, to achieve a DOPC:DSPC:cholesterol 2:1:1 mol:mol:mol ratio. The resulting organic solution was diluted with a CH_2Cl_2:MeOH 2:1 solution to reach a final 10 mg mL^{-1} lipid concentration. The organic solution was dried under reduced pressure using a rotary evaporator. The dried lipid film was heated to 55 ˚C and pre-heated (55 °C) HBSS buffer was added dropwise while stirring, to achieve a 0.02 mg mL^{-1} lipid concentration. The mixture was stirred gently (100 rpm) until it became clear, and then was subjected to 21 successive extrusions through a polycarbonate membrane (0.2 μm pore size). The resulting liposome suspension was analysed by DLS for particle size analysis (Figure S17).

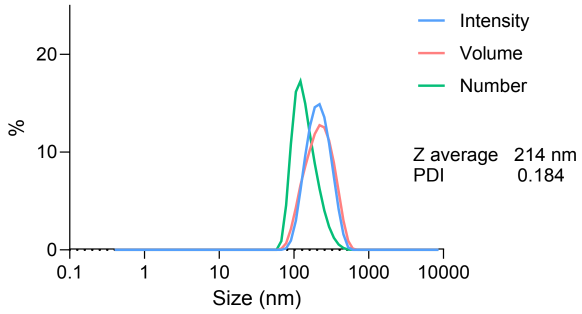

Figure S17. Dynamic light scattering (DLS) particle size analysis of liposomes (DOPC:DSPC:cholesterol 2:1:1 mol:mol:mol) employed to form immobilisied bilayers for the SPR studies. Data are shown as intensity, volume and number distributons (%).

The loading process was conducted in three successive steps:

Surface wash: the L1 chip surface was cleaned with repeated (at least 2) injections of 10 µL of 20 mM CHAPS detergent solution.

Liposome loading: the liposome suspension (prepared as described above) was injected at a low flow rate (5 µL min^{-1}) at 0.5 mM lipid concentration. Adsorption was monitored by following an increase in Response index Units (RiU), which plateaued as the surface coverage approached completion. Previous studies by Karlsson[9] and Kamimori[10] suggest that values around 5000 RiU are indicative of formation of a supported bilayer on the sensor surface. Accordingly, the liposome suspension (0.02 mg/mL in 10 mM HBSS) was then injected on the L1 chip at 5 µL min^{-1}, until a stable baseline at 5600 RiU was reached. Bilayer immobilisation was found to be complete within a few minutes.

Liposomal surface cleaning: Regeneration of the sensor chip surface was performed using a long pulse of the buffer solution (10 mM HBSS) at high flow rate (50 µL min^{-1}, 420 sec).

Table S3. Kinetic parameters obtained from SPR analysis for the interaction between the tested polymers and L1-immobilised bilayers.

**Polymer**	**k_a (M^{-1} s^{-1})[a]**	**k_d (s^{-1})[b]**	**K_D (M)[c]**	**R_{max} (RiU)[d]**
Chol-$(HEA)_{58}$	3.71×10^{6}	3.16×10^{-3}	8.50×10^{-10}	1996
Chol-$(HEA)_{70}$	3.51×10^{6}	3.12×10^{-3}	8.88×10^{-10}	1706
Chol-$(HEA)_{108}$	1.64×10^{7}	5.33×10^{-3}	3.26×10^{-10}	2456
DOPE-$(HEA)_{80}$	2.19×10^{3}	3.31×10^{-5}	1.51×10^{-8}	1718
PABTC-$(HEA)_{63}$	-	-	-	-

[a] association rate constant, [b] dissociation rate constant, [c] equilibrium dissociation constant and [d] maximum response.

Solid-state NMR analysis of multilamellar lipid vesicles

The selected polymers were solubilised or suspended in the minimum amount of deionised water and added to solid dimyristoyl phosphatidylcholine (DMPC:polymer 10:1 molar ratio); the mixture was stirred until an almost clear suspension was obtained. The mixture was subjected to 8 freeze-thaw cycles to produce multilamellar vesicles, then centrifuged for 20 minutes at 1500 rpm. The supernatant was removed, and the pellet collected and transferred to the solid-state NMR probe. All solid-state NMR experiments were performed with a Varian 400 MHz VNMRS spectrometer equipped with a 4 mm magic angle spinning (MAS) NMR probe. ^{31}P and ^{13}C NMR spectra were referenced externally with 10% H_3PO_4 and adamantane, respectively.

^{31}P wideline NMR was performed at 28 ˚C using Hahn echo sequence with 100 kHz π/2 pulse and 12 μs delays. Spectra were acquired with 20 ms acquisition time with a recycle delay of 5 s and 2048 transients averaged.

^{13}C Magic Angle Spinning (MAS) NMR was performed at MAS frequency of 5 kHz and at 32 ˚C, for both cross polarisation and direct excitation experiments. Direct excitation experiments were done with a single 100 kHz π/2 before acquisition, followed by 125 ms acquisition under 60 kHz SPINAL-64 to remove heteronuclear dipolar couplings. The recycle delay was 5 s, and spectra were obtained after average of 2048 transients.

Cross polarisation was performed with an initial 120 kHz π/2 1H excitation pulse, followed by 3.5 ms of 45 kHz Harmann-Hahn contact time for magnetisation transfer to 13C. Spectra were recorded under 60 kHz SPINAL-64 scheme over 125 ms of acquisition time. The recycle delay was 3.5 s, and spectra were obtained by averaging 2048 transients.

Molecular modelling of the interactions of Chol-HEA$_n$ homopolymers with a DPPC bilayer membrane

All simulations were performed using AMBER 2028–31. For atomistic modelling purposes, Chol-(HEA)$_n$ polymers were divided into cholesterol, linker, HEA repeating units (both enantiomers) and terminal units, with appropriate capping groups. Models for each were built using Chimera[11], and then unit parameter files generated using AMBER's Antechamber module[12].

Cytotoxicity of polymers synthesised in this work: PrestoBlue™ and LDH assays.

Polymer toxicity was investigated by lactate dihydrogenase (LDH) assays, which detects cell membrane damage by detecting the LDH enzyme released from the cytoplasm[13, 14] and by using PrestoBlue™, a resazurin-based assay that estimates the viability of cells by estimating their metabolic activity.[15, 16]

Caco-2 cells were seeded at concentration $1*10^4$ per well in 96-well plates (black) and cultured for 24 hours. The medium (DMEM) was removed and replaced with solutions of selected polymers in HBSS and cells were incubated for either 30 min or 24 h, in phenol-free medium. Following incubation with polymers, 50 µL of supernatant from each well were transferred to another 96-well plate, LDH solution was added to each well and after 25 min incubation the absorbance at λ=490 nm was measured. Relative LDH release was calculated through Equation S2, with the negative control taken as untreated cells, and positive control as total cell lysis (cells treated with a 1% Triton X-100 solution for 30 minutes).

$$Relative\ LDH\ release = \left(\frac{(\mathrm{A}x - \mathrm{A}(Negative\ control))}{(\mathrm{A}(Positive\ control) - \mathrm{A}(Negative\ control))}\right) \times\ 100$$

Equation S2. Calculation of relative LDH release. "A" represents absorbance at λ = 490 nm; A_x represents absorbance of polymer-treated cells.

The solution remaining in the original plate was washed off with PBS, PrestoBlue™ (Thermofisher) 10% solution (in phenol red-free medium) was added, and after 60 min incubation fluorescence was measured at λ_{em} = 600 nm (λ_{ex} =560 nm). The relative metabolic activity was calculated through Equation S3, with the negative control as untreated cells, and negative control as total cell lysis (cells treated with a 1% Triton X-100 solution for 30 minutes):

$$Relative\ metabolic\ activity = \left(\frac{(\mathrm{I}x - \mathrm{I}(Positive\ control))}{(\mathrm{I}(Negative\ control) - \mathrm{I}(Positive\ control))}\right) \times\ 100$$

Equation S3. Calculation of relative metabolic activity, PrestoBlue™ assay. I = fluorescence emission intensity following excitation at λ_{ex} = 560 nm and emission at λ_{em} = 600 nm.

The 96-well plates were analysed with a Spark 10M Plate Reader (Tecan) and analysed with SparkControl 2.1 software, then further elaborated with Prism 8.4.2 (GraphPad). The measurement was run at 37 °C, with λ_{ex} = 560 nm and λ_{em} = 600 nm for PrestoBlue™ and absorbance at λ = 490 nm for LDH.

Initially, LDH and PrestoBlue assays were carried out for a range of Chol- (Figure S14), PABTC- (Figure S15) and DOPE-polymers (Figure S16).

24 h incubation time

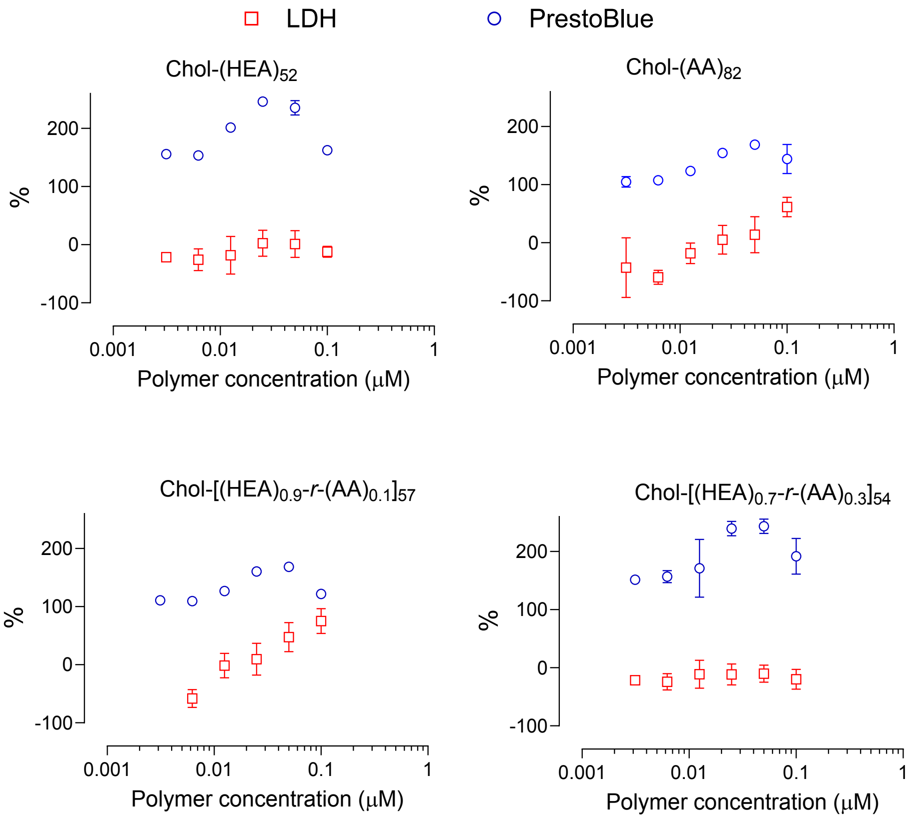

Figure S18. Effect of Chol-terminated polymers in Caco-2 cells metabolic activity (PrestoBlue assay) and cell membrane integrity (LDH assay). The assays were performed on Caco-2 cells, after incubation with polymers for 24 hours. 100% and 0% membrane damage refer to cells treated with a 1% Triton X-100 solution, and untreated cells, respectively (n = 9, error bars expressed as SD).

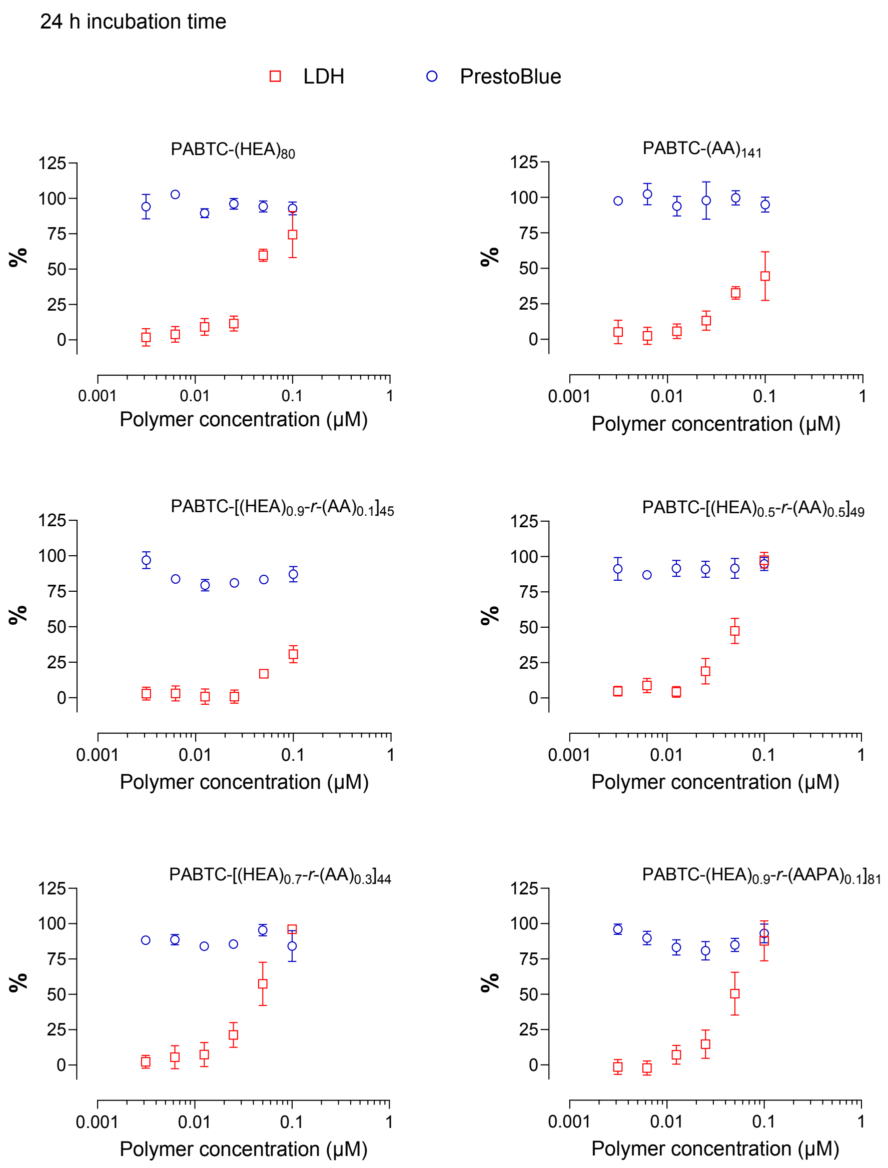

Figure S19. Effect of PABTC-terminated polymers in Caco-2 cells metabolic activity (PrestoBlue assay) and cell membrane integrity (LDH assay). The assays were performed on Caco-2 cells, after incubation with polymers for 24 hours. 100% and 0% membrane damage refer to cells treated with a 1% Triton X-100 solution, and untreated cells, respectively (n = 9, error bars expressed as SD).

24 h incubation time

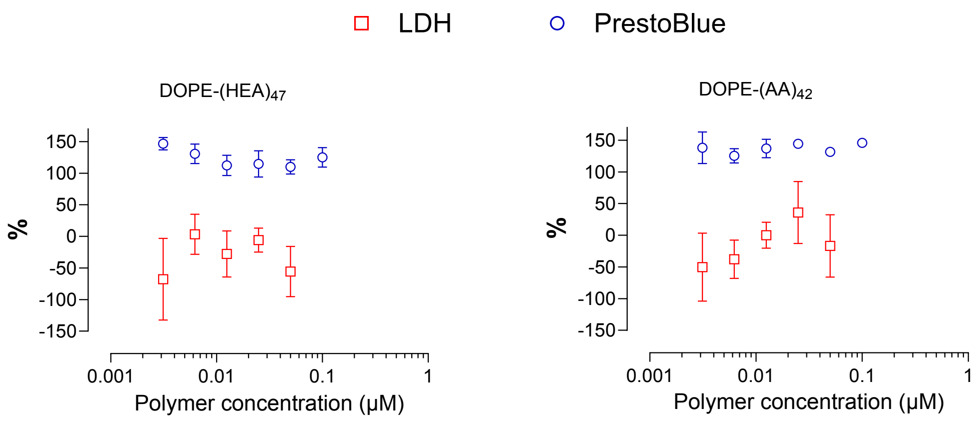

Figure S20. Effect of DOPE-terminated polymers in Caco-2 cells metabolic activity (PrestoBlue assay) and cell membrane integrity (LDH assay). The assays were performed on Caco-2 cells, after incubation with polymers for 24 hours. 100% and 0% membrane damage refer to cells treated with a 1% Triton X-100 solution, and untreated cells, respectively (n = 9, error bars expressed as SD).

The membrane fluidity laurdan assay described in this work (Figure 1) were carried out by incubating Caco 2 cells with polymers for 30 minutes. This, to rule out that some of the effect observed in the laurdan assay where due membrane damage induced by the polymers used in this work, LDH and presto blue assays were carried out using Chol-[(HEA)$_{0.9}$-*r*-(AA)$_{0.1}$]$_{57}$ and Chol-[(HEA)$_{0.7}$-*r*-(AA)$_{0.3}$]$_{54,}$ the polymers that showed the highest increase in cell metabolism and LDH levels in the 24 hours experiments. Results show that incubation of Caco-2 cells with Chol-[(HEA)$_{0.9}$-*r*-(AA)$_{0.1}$]$_{57}$ and Chol-[(HEA)$_{0.7}$-*r*-(AA)$_{0.3}$]$_{54}$ polymers for 30 minutes did not induce changes in neither in membrane integrity (LDH assay) nor in cell metabolism (PrestoBlue assay) (Figure S17).

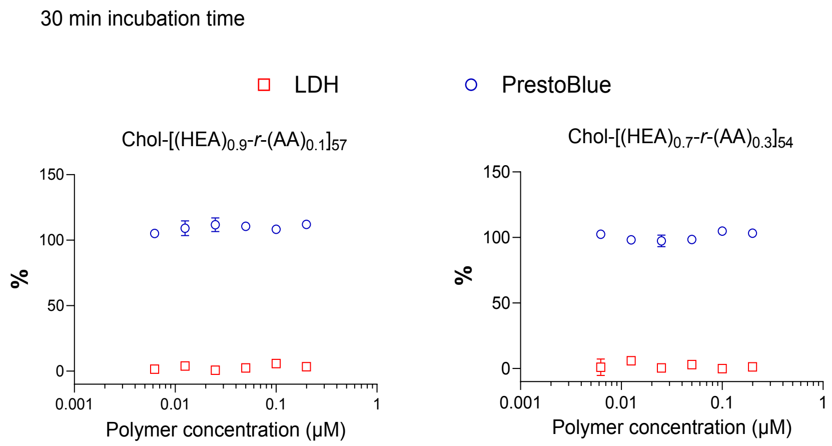

Figure S21. Effect of selected Chol-terminated polymers in Caco-2 cells metabolic activity (PrestoBlue assay) and cell membrane integrity (LDH assay). The assays were performed on Caco-2 cells, after incubation with polymers for 30 minutes. 100% and 0% membrane damage refer to cells treated with a 1% Triton X-100 solution, and untreated cells, respectively (n = 9, error bars expressed as SD).

Finally, an LDH test was carried out on Caco-2 cells incubated with Chol- and PABTC-polymers – DOPE polymers did not induce LDH release – for 30 min, at 0.1 μM, the highest concentration used for the previous LDH experiments, and also the concentration of the polymers used for the laurdan membrane fluidity assay (Figure 1). Under these conditions, only minimal to no LDH release was detected (Figure S21).

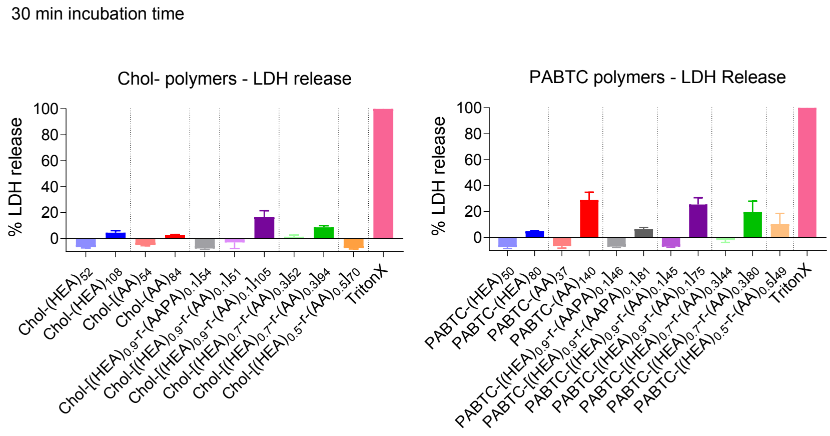

Figure S22. Effect of Chol- and PABTC-terminated polymers in Caco-2 cell membrane integrity (LDH assay). The assays were performed on Caco-2 cells, after incubation with polymers for 30 min at a 0.1 μM concentration. 100% and 0% membrane damage refer to cells treated with a 1% Triton X-100 solution, and untreated cells, respectively (n = 6, error bars expressed as SD).

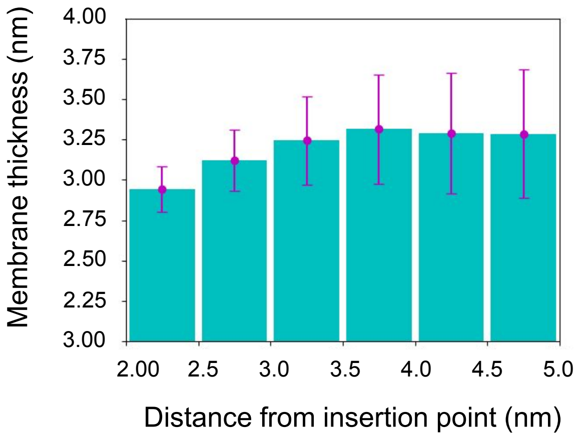

Figure S22. Calculated mean membrane thickness (minimum distance between phosphorus atoms in the two leaflets) as a function of radial (x,y) distance from the inserted cholesterol for the insertion, using data from the last 300ns of the Chol-(HEA)$_{58}$ simulation.

NMR spectra.

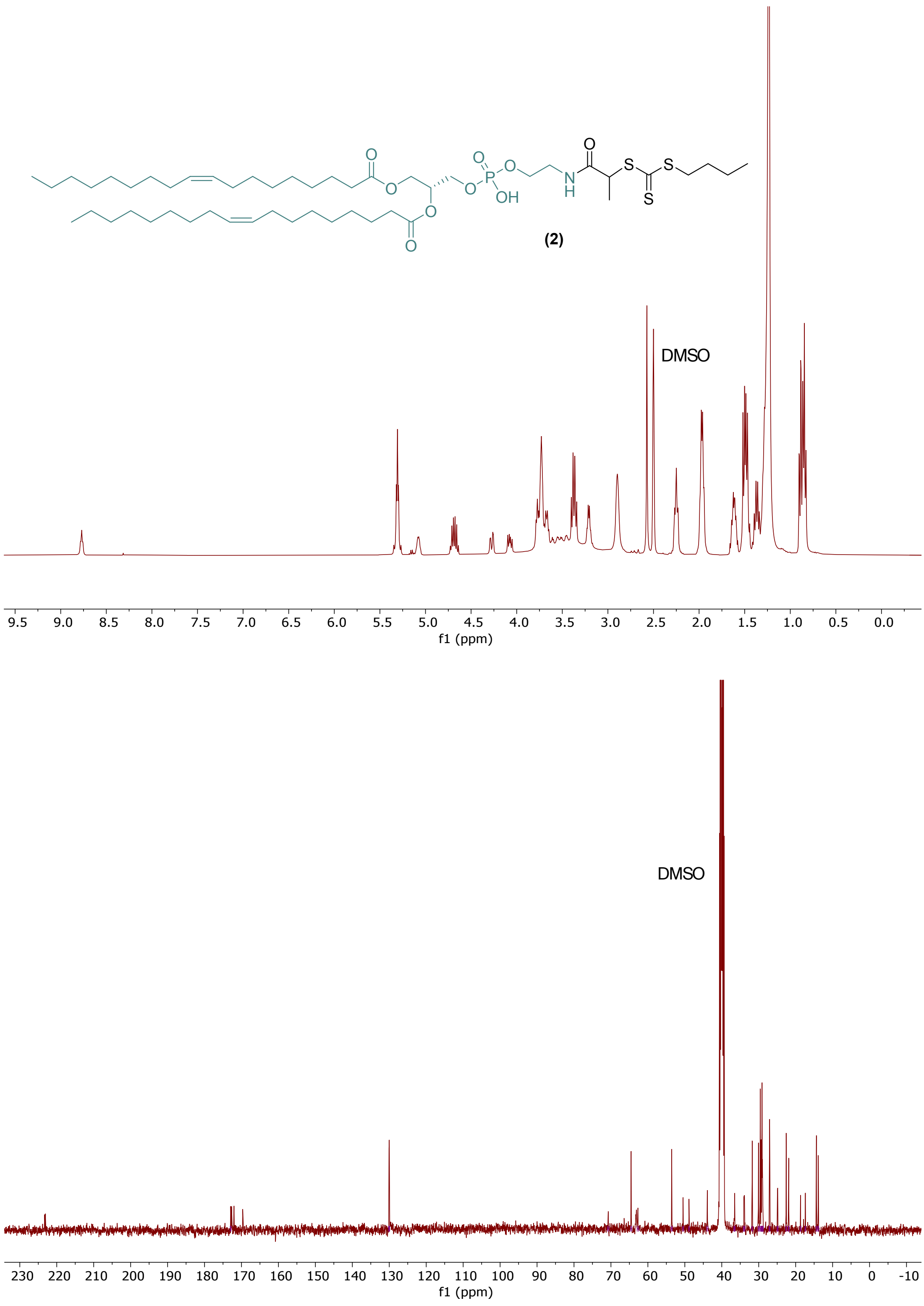

Figure S23. ^1H and ^{13}C NMR spectrum of DOPE RAFT agent **(2)** in DMSO-d6.

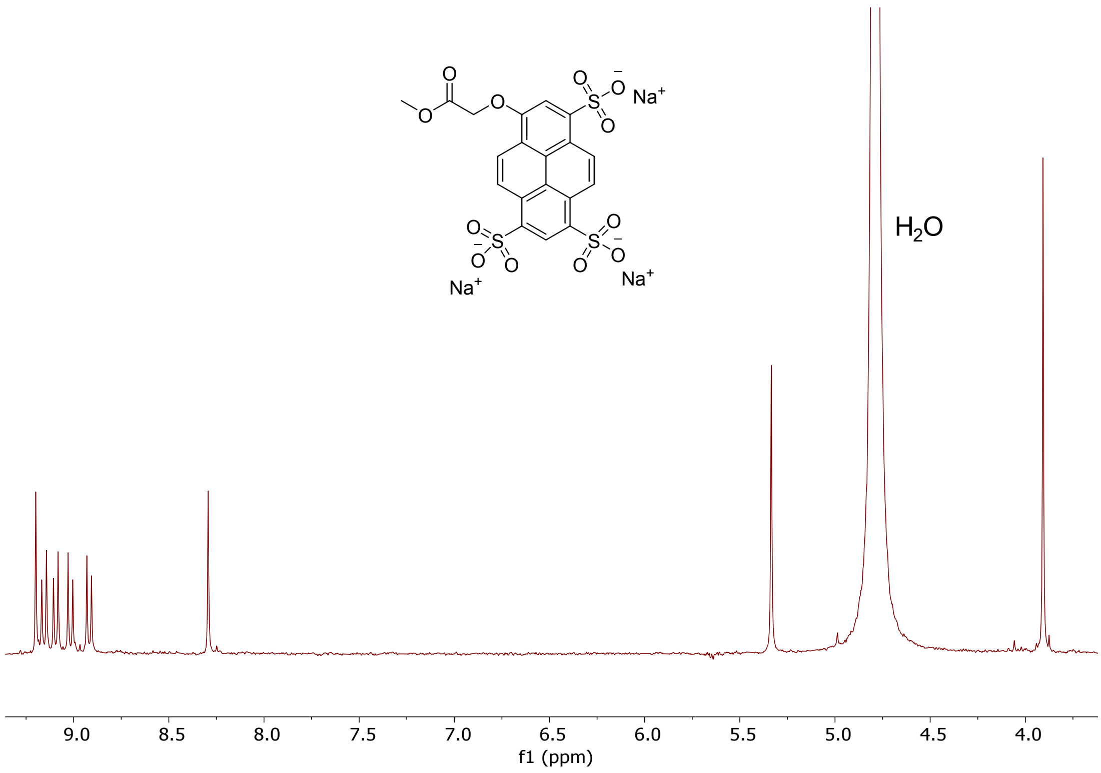

Figure S24. ^{1}H NMR spectrum of Pyranine methyl ester **(4)** in D_2O.

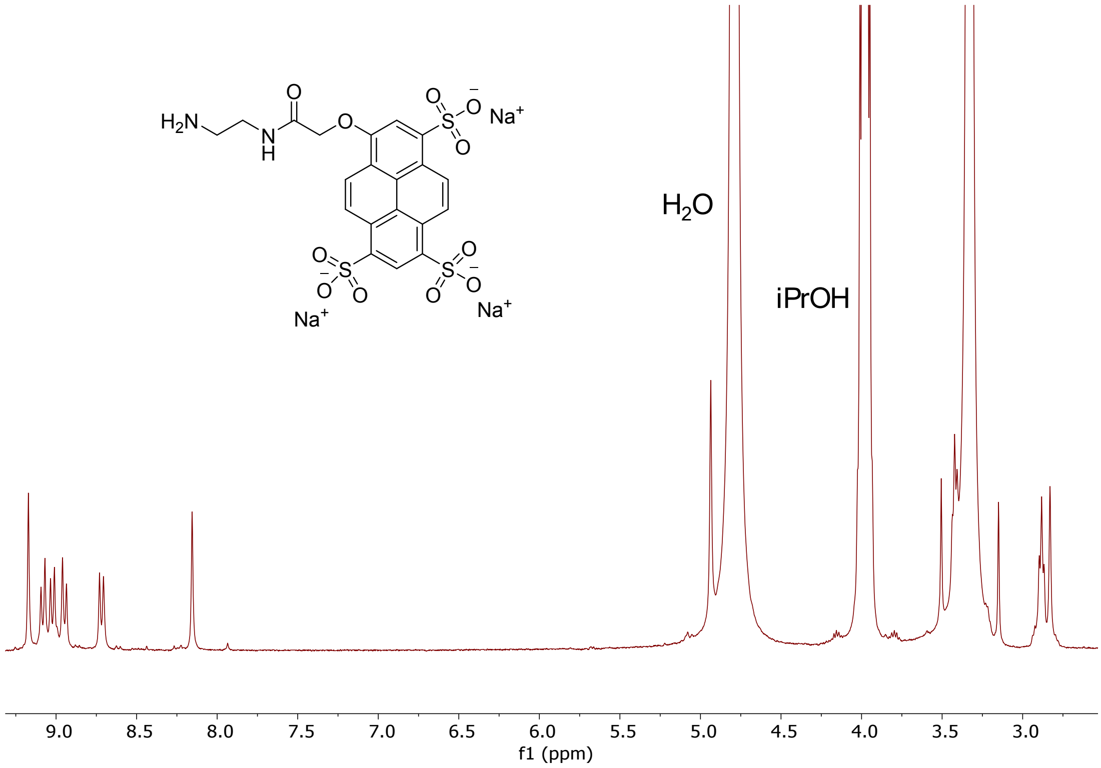

Figure S25. ^{1}H NMR spectrum of Pyranine ethylenediamine **(5)** in D_2O.

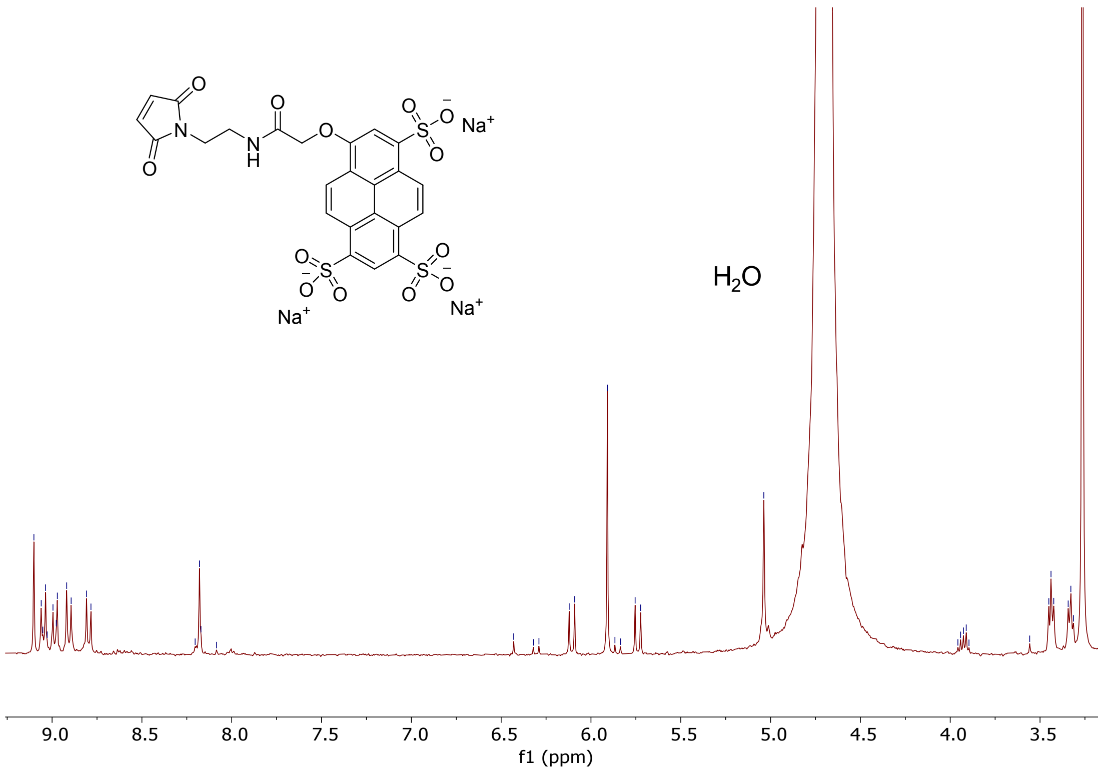

Figure S26. ^{1}H NMR spectrum of MalPyr Blue **(6)** in D_2O.

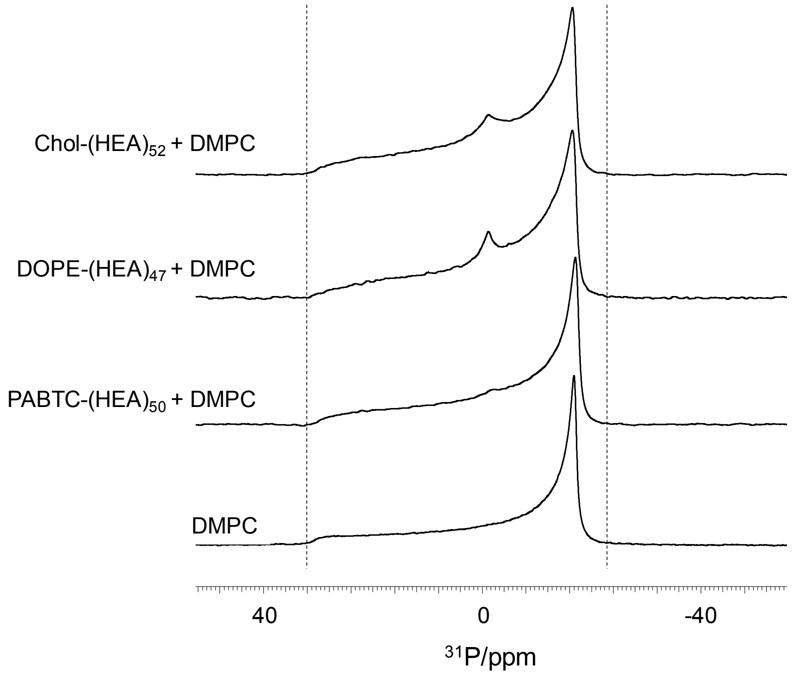

Figure S27. ^{31}P MAS wideline spectra of DMPC MLVs treated with polymers. A) DOPE-(HEA)$_{47}$; B) Chol-(HEA)$_{52}$; C) PABTC-(HEA)$_{50}$; D) DMPC MLVs. The additional isotropic peak around 0 ppm indicates presence of non-bilayer structures, therefore that the polymer interaction with the bilayer is likely causing membrane instability and membrane shedding.

References.

1. R. Catania, F. Mastrotto, C. J. Moore, C. Bosquillon, F. H. Falcone, A. Huett, G. Mantovani and S. Stolnik, *Advanced Therapeutics*, 2021, **4**, 2100168.
2. C. J. Ferguson, R. J. Hughes, D. Nguyen, B. T. T. Pham, R. G. Gilbert, A. K. Serelis, C. H. Such and B. S. Hawkett, *Macromolecules*, 2005, **38**, 2191-2204.
3. M. Danial, C. M. N. Tran, K. A. Jolliffe and S. Perrier, *Journal of the American Chemical Society*, 2014, **136**, 8018-8026.
4. A. Dominguez, A. Fernandez, N. Gonzalez, E. Iglesias and L. Montenegro, *J. Chem. Educ.*, 1997, **74**, 1227.
5. R. J. Cavanagh, P. A. Smith and S. Stolnik, *Mol. Pharm.*, 2019, **16**, 618-631.
6. K. Akashi, H. Miyata, H. Itoh and K. Kinosita, *Biophys. J.*, 1996, **71**, 3242-3250.
7. S. Vemuri and C. T. Rhodes, *Pharm. Acta Helv.*, 1995, **70**, 95-111.
8. F. Olson, C. A. Hunt, F. C. Szoka, W. J. Vail and D. Papahadjopoulos, *Biochim. Biophys. Acta*, 1979, **557**, 9-23.
9. O. P. Karlsson and S. Löfås, *Anal. Biochem.*, 2002, **300**, 132-138.
10. H. Kamimori, J. Blazyk and M.-I. Aguilar, *Biological and Pharmaceutical Bulletin*, 2005, **28**, 148-150.
11. E. F. Pettersen, T. D. Goddard, C. C. Huang, G. S. Couch, D. M. Greenblatt, E. C. Meng and T. E. Ferrin, *J. Comput. Chem.*, 2004, **25**, 1605-1612.
12. J. Wang, W. Wang, P. A. Kollman and D. A. Case, *Journal of Molecular Graphics and Modelling*, 2006, **25**, 247-260.
13. T. Decker and M.-L. Lohmann-Matthes, *J. Immunol. Methods*, 1988, **115**, 61-69.
14. S. M. Smith, M. B. Wunder, D. A. Norris and Y. G. Shellman, *PLoS One*, 2011, **6**, e26908.
15. M. Xu, D. J. McCanna and J. G. Sivak, *Journal of Pharmacological and Toxicological Methods*, 2015, **71**, 1-7.
16. M. Sonnaert, I. Papantoniou, F. P. Luyten and J. I. Schrooten.
